# Effects of exercise on cognitive function in children and adolescents with overweight or obesity: a systematic review and meta-analysis of randomized controlled trials

**DOI:** 10.3389/fpubh.2025.1694170

**Published:** 2025-12-12

**Authors:** Shi Li, Mengyan Wu, Jie Hu, Jingfeng Chen, Bichen Xiong, Deyu Jiao, Pengbo Ren, Hongwei Liu, Haixia Fan

**Affiliations:** 1 Department of Sports, Wuxi City College of Vocational Technology, Wuxi, Jiangsu, China; 2 Department of Sports, Suzhou City University, Suzhou, Jiangsu, China; 3 Guangzhou Sport University, Guangzhou, Guangdong, China; 4 Department of Health and Physical Education, The Education University of Hong Kong, Hong Kong, China; 5 Department of Police Sports, Zhejiang Police College, Hangzhou, Zhejiang, China; 6 High School Affiliated to Nanjing Normal University, Nanjing, Jiangsu, China; 7 Department of Sports, Changzhou University Huaide College, Jingjiang, Jiangsu, China; 8 Department of Neurology, Taiyuan City Central Hospital, The Ninth Clinical Medical College of Shanxi Medical University, Taiyuan, Shanxi, China; 9 Department of Sleep Center, First Hospital of Shanxi Medical University, Taiyuan, Shanxi, China

**Keywords:** cognitive function, exercise, meta-analysis, childhood, adolescent

## Abstract

**Background:**

Exercise is a non-pharmacological strategy for enhancing cognitive function among various populations. The aim of our systematic review was to synthesise evidence of the effects of exercise on cognitive function in children and adolescents with overweight or obesity.

**Methods:**

A comprehensive search of four databases (Web of Science, PubMed, Scopus, EMBASE was conducted from each database inception through 1 April, 2025. This review investigates the impact of exercise intervention on the cognitive function of children and adolescents with overweight or obesity through randomized controlled trials (RCTs). The quality of the included studies was evaluated using the Cochrane Risk of Bias Tool 2, and the treatment effects were analyzed through random-effects or fixed-effects models, with Hedges’ g serving as the metric for effect size estimation. I^2^ statistics assessed heterogeneity, and leave-one-out analysis verified result stability. Subgroup analyses were conducted based on the FITT principle, including exercise frequency, intensity, and time.

**Results:**

The meta-analysis of 15 RCTs, including 1,210 children and adolescents with overweight or obesity revealed that exercise significantly improved executive function (*g* = 0.39, 95% CI:0.12 to 0.66, *p* = 0.0043). No significant effects were observed for other aspects of attention and memory (*p*>0.05). Subgroup analysis identified that exercise with a frequency of >3 sessions per week (g = 0.39, *p* = 0.0257), moderate-to-vigorous intensity (*g* = 0.50, *p* = 0.0132), exercise interventions ≥10 weeks (*g* = 0.41, *p* = 0.0184), single session duration >30 min (*g* = 0.41, *p* = 0.0139), and weekly exercise volume > 120 min (*g* = 0.28, *p* = 0.00236) had higher effect sizes in improving executive function in children and adolescents with overweight or obesity. Subgroup analyses revealed that exercise frequencies <5 sessions/week (*g* = 0.98), single-session durations ≥40 min (*g* = 0.60), weekly volumes ≥120 min (*g* = 0.60), and intervention periods >15 weeks (*g* = 0.98) significantly enhanced attention (*p* < 0.05).

**Conclusion:**

This study demonstrates that exercise interventions significantly improve executive function and attention in children and adolescents with overweight or obesity. In particular, moderate-intensity aerobic exercise performed more than three times per week, for over 30 min per session, and sustained for at least 10 weeks appears to represent the optimal training parameters for improving executive function. These findings support the use of exercise as a cognitive enhancement strategy. However, larger-scale studies are needed to confirm its effects on other cognitive domains.

**Systematic review registration:**

https://www.crd.york.ac.uk/PROSPERO/view/CRD420251078572, Identifier: CRD420251078572.

## Graphical abstract

**Figure:**
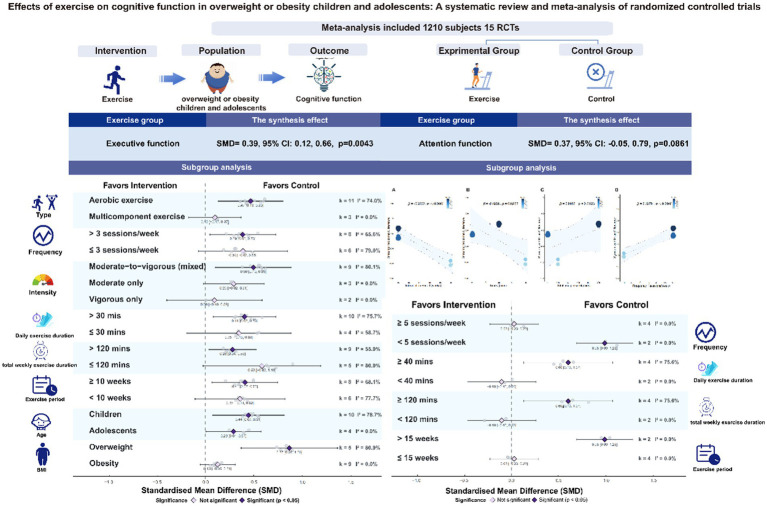

## Highlights

- Aerobic exercise intervention demonstrably enhances executive function in children and adolescents with overweight or obesity.
- The most effective exercise regimen for improving executive function involves moderate-to-vigorous intensity aerobic exercise performed >3 times/week, >30 minutes/session, with a weekly volume >120 minutes, sustained for ≥10 weeks.
- Although findings on the effects of exercise on attention remain inconsistent, positive outcomes have been observed with interventions conducted < 5 times per week, for at least 40 minutes/session, totaling ≥120 minutes/week, and sustained for over 15 weeks.

## Introduction

Childhood and adolescent overweight and obesity, defined as excessive fat accumulation with a body mass index (BMI) at or above the 85th percentile, have become a pressing global public health issue (1–4). According to population surveys and analyses of children aged 5 to 19 up to 2016, approximately 50 million girls and 74 million boys worldwide were diagnosed with obesity (5). A recent systematic review and meta-analysis has shown that the prevalence rates of overweight and obesity among global children and adolescents are as high as 8.5 and 14.8%, respectively (6). Notably, between 2020 and 2035, the obesity rate among boys globally is projected to increase by 10 to 20%, while that among girls will rise by 8 to 18% (7). Beyond elevating the risk of metabolic disorders, childhood and adolescent obesity exerts detrimental effects on cognition at both physiological and psychological levels (8, 9). For example, researchers have found in three species (drosophila, mice, and humans) that adipose tissue influences cognitive functions (such as memory and learning ability) through the expression of genes related to axon guidance, nervous system development, and inflammation. Additionally, the obesity state leads to a decline in the cognitive ability of living organisms (10). Meanwhile, epidemiological surveys reveal that obesity-related body dissatisfaction, depressive symptoms, and other psychopathological conditions are significantly associated with cognitive dysfunction in childhood and adolescent (11–14). Emerging evidence further suggests a bidirectional relationship between obesity and cognitive function, wherein cognitive impairments may exacerbate binge-eating behaviors and perpetuate weight gain (15). These findings underscore the urgent need for effective interventions to mitigate obesity-related cognitive dysfunction in children and adolescents.

Over recent decades, numerous clinical and retrospective studies have demonstrated that physical exercise can improve cognitive function to varying degrees in healthy or special populations of children and adolescents. Improvements have been observed across cognitive subdomains such as executive function, cognitive flexibility, and attention, including among individuals with autism spectrum disorder, neurodevelopmental disorders, or attention-deficit/hyperactivity disorder (16–20). Given its effective non-pharmacological therapeutic effects, accessibility, and cost-effectiveness, physical exercise has been recognized as a critical strategy for managing in children and adolescents with overweight and obesity (21–23). Current limited research suggests potential benefits of exercise interventions for the cognitive function of children and adolescents with overweight or obesity. For instance, three systematic reviews and meta-analyses reported that exercise interventions significantly enhanced executive function (e.g., cognitive flexibility) in children and adolescents with overweight or obesity, though no significant effects were observed for attention (24–26). In contrast, a recent meta-analysis focusing on aerobic exercise shows that aerobic exercise failed to significantly enhance cognitive flexibility in children with overweight or obesity (27). Additionally, physical activity interventions have been reported to potentially exert more positive effects on the executive function of overweight or obesity children and adolescents than on other cognitive domains (e.g., metacognition and academic performance) (28). These inconsistencies may reflect heterogeneity in inclusion criteria (e.g., exercise modality and participant characteristics) and the limitations of existing systematic reviews. Meanwhile, previous studies have exhibited methodological and experimental constraints. For instance, the aforementioned studies typically compared only one exercise characteristic or one type of cognitive function in children and adolescents with overweight or obesity, lacking comprehensive assessments of more exercise variables (e.g., exercise frequency and duration) and multi-dimensional cognitive subdomains (29–33). These gaps underscore the need for integrative and systematic research examining how exercise influences diverse cognitive functions in children and adolescents with overweight or obesity.

This systematic review and meta-analysis aim to explore the effects of long-term exercise interventions on the cognitive function of children and adolescents with overweight or obesity. We further investigate the differential impacts of various exercise variables (such as type, duration, and intervention cycle) on cognitive subdomains through subgroup analyses.

## Methods

### Search strategy

The systematic review was conducted according to the Preferred Reporting Items for PRISMA guidelines and the Cochrane Collaboration Handbook (34). The current review was prospectively registered with PROSPERO (CRD: 420251078572). We conducted a comprehensive literature search to identify studies on effects of exercise on cognitive function in overweight or obesity children and adolescents. We searched four electronic databases (Web of Science, PubMed, Scopus, EMBASE) from the year of their inception up to 18 April 2025. Our search strategy was modified in accordance with each of the databases (e.g., using MeSH terms where possible), and included the following keywords: exercise (e.g., training, Physical activity, resistance exercise and aerobic exercise), executive functions (e.g., inhibition, working memory, cognitive flexibility), and overweight/obesity populations (e.g., overweight children, obesity adolescents). The detailed search strategies are provided in Supplementary Material S1.

### Study selection

The retrieved articles were imported into EndNote 8.0 software for de-duplication. Two investigators (LS and HL) independently performed all records selection processes, including title/abstract screening and full-text assessment using a standardized form.

Disagreements between the two authors were resolved via discussion and arbitration by a third researcher (FH). The inclusion criteria were: (1) school-aged children and adolescents who were overweight (≥85th percentile BMI for age and sex) or obesity (≥95th percentile BMI for age and sex) and aged 5–18 years (35); (2) participants receiving physical exercise intervention, including aerobic exercise, resistance exercise, mind–body exercise, and multi-component exercise; (3) Outcome Measures: At least one assessment related to cognitive function, such as executive function (i.e., inhibition, working memory, cognitive flexibility). (4) peer-reviewed articles published in English; (5) RCT design; (6) comparison with a non-intervention control group (e.g., waiting list, treatment as usual, or health education). Exclusion criteria were: (1) Studies involving treatments for cognitive function combined with exercise (e.g., dietary or sleep interventions); (2) studies with data that could not be extracted or were not clearly reported.

### Data extraction and bias assessment

For each eligible study, two independent researchers (LS and HL) extracted data on, the following: first author, publication year, study location, sample size, participant age, gender distribution, intervention characteristics, and cognitive function outcome measures. When the necessary data were unavailable, the original authors were contacted by email. Discrepancies in data extraction were resolved through discussion. Two independent reviewers (LS and HL) assessed the methodological quality of each eligible RCT using the revised Risk of Bias 2 (RoB 2) tool. The assessment covered five key domains: (1) randomization process, (2) deviations from intended interventions, (3) missing outcome data, (4) measurement of outcomes, and (5) selection of reported results. Based on the RoB 2 criteria, studies were categorized as “low risk of bias” (i.e., all domains judged as low risk), “some concerns” (i.e., at least one domain with some concerns), or “high risk of bias” (i.e., at least one domain with high risk or multiple domains with some concerns). In addition, publication bias was assessed through both funnel plot analysis (36) and the Egger’s test (37). Any discrepancies were resolved through consensus-based discussion.

### Data synthesis and analysis

All meta-analytic calculations were performed using R software (version 4.5.1) with the “meta” and “metafor” packages (38, 39). Following the intervention, the alterations in outcomes were analysed employing either a fixed-effects or a random-effects model (40). A forest plot was generated to visualise the results of cognitive function comparisons between the exercise and control groups. Hedges’ g and 95% confidence intervals were computed using post-intervention data, namely the means and standard deviations (SD) of cognitive function in each group. The correction factor for small-sample bias was incorporated into the calculation of Hedges’ g to ensure the accuracy of effect size estimation. When unavailable, these were derived from alternative statistics (e.g., standard errors, confidence intervals, or *p*-values). In studies incorporating shared control groups, the sample size of the control group was reduced by half to mitigate the occurrence of unit-of-analysis errors (41–43). The magnitude of the effect size was categorized as small (0.2 ≤ *g* < 0.5), medium (0.5 ≤ *g* < 0.8), or large (*g* ≥ 0.8), with statistical significance at *α* < 0.05 (two-tailed). Study heterogeneity was assessed using I^2^ statistic (low: <25%, moderate: 25–50%, and high: >50%) (44). The choice of model was contingent upon the degree of heterogeneity: a random-effects model was used for I^2^ values greater than 50%, and a fixed-effects model for *I*^2^ values of 50% or less (44). Sensitivity analyses were conducted using the leave-one-out method to examine the effect of individual studies on pooled effects. The exclusion of each study was carried out sequentially, and the meta-analysis was then recomputed to verify the stability of the results. Subgroup analyses were conducted to determine potential moderating effects of exercise frequency (≤3 or >3 sessions/week), exercise type (aerobic exercise, resistance exercise, or multi-component exercise), session duration (≤30, >30 min), intervention length (≤10 or >10 weeks), and exercise intensity (moderate-to vigorous, moderate only, vigorous only) on outcomes. Additionally, subgroup analyses were conducted based on age and BMI.

## Results

### Search selection

**Figure 1:**
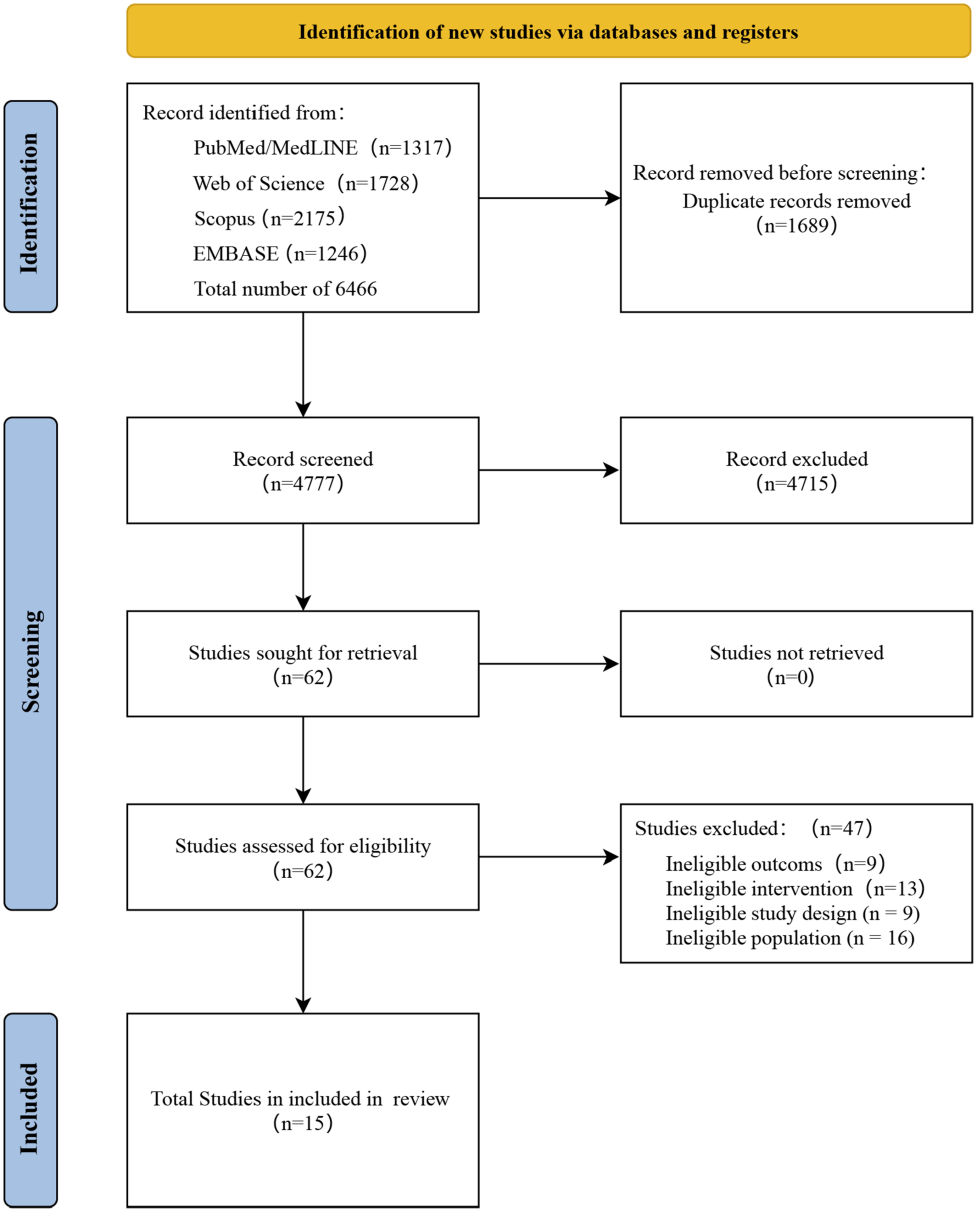
Flow diagram of systematic literature search selection. RCT randomized controlled trial.

A total of 6,466 literatures were initially identified from 4 databases. After removing duplicates (*n =* 1,689), 4,777 studies were subjected to a review process based on titles and abstracts. Following this initial review, 62 studies were selected for a comprehensive full-text assessment for eligibility. A total of 15 RCTs (randomized controlled trials) were included (45–59). The study selection process and literature screening are illustrated in Figure 1.

### Characteristics of studies

The included studies involved between 36 (48) and 171 (58) participants, with mean ages ranging from 8 (49) to 14.6 years (50) (Table 1). China contributed the largest number of studies (*n =* 7), followed by the USA (*n =* 4), Spain (*n =* 2), Italy (*n =* 1), and Denmark (*n =* 1). Exercise interventions lasted from one (48, 50) to 39 weeks (49). Exercise session length ranged from 20 min (46, 58) to 300 min (52), with 40 min being the most commonly implemented duration (46, 51, 54, 56). Intervention frequency ranged from once per week (48, 50) to daily sessions (55), with 5 days per week being the most frequency adopted (46, 49, 56). Exercise modality differed across studies, through most interventions employed aerobic exercise alone (*n =* 12), combined aerobic and strength training (*n =* 1), or multi-component exercise (*n =* 2). The intensity applied was also heterogeneous, with moderate-to-vigorous (mixed) intensity being the most common (*n =* 9), while the remaining studies used moderate-only (*n =* 3) or vigorous-only (*n =* 2) intensity. The groups included as controls were primarily wait-list and (conventional) control groups. Notably, none of the studies monitored physical activity behaviors in the control groups. Study characteristics are summarized in Table 1.

**Table 1:** Study inclusion criteria based on PICOS strategy.

Parameter	Inclusion criteria
Population	Children and adolescents aged 5–18 years with overweight (≥85^th^ percentile BMI) or obesity (≥95^th^ percentile BMI).
Intervention	Exercise
Comparator	Non-intervention control group (e.g., waiting list, treatment as usual, or health education)
Outcome	Cognitive function (executive function, attention, memory)
Study design	Randomized control trials

### Methodological quality and risk of bias

**Figure 2:**
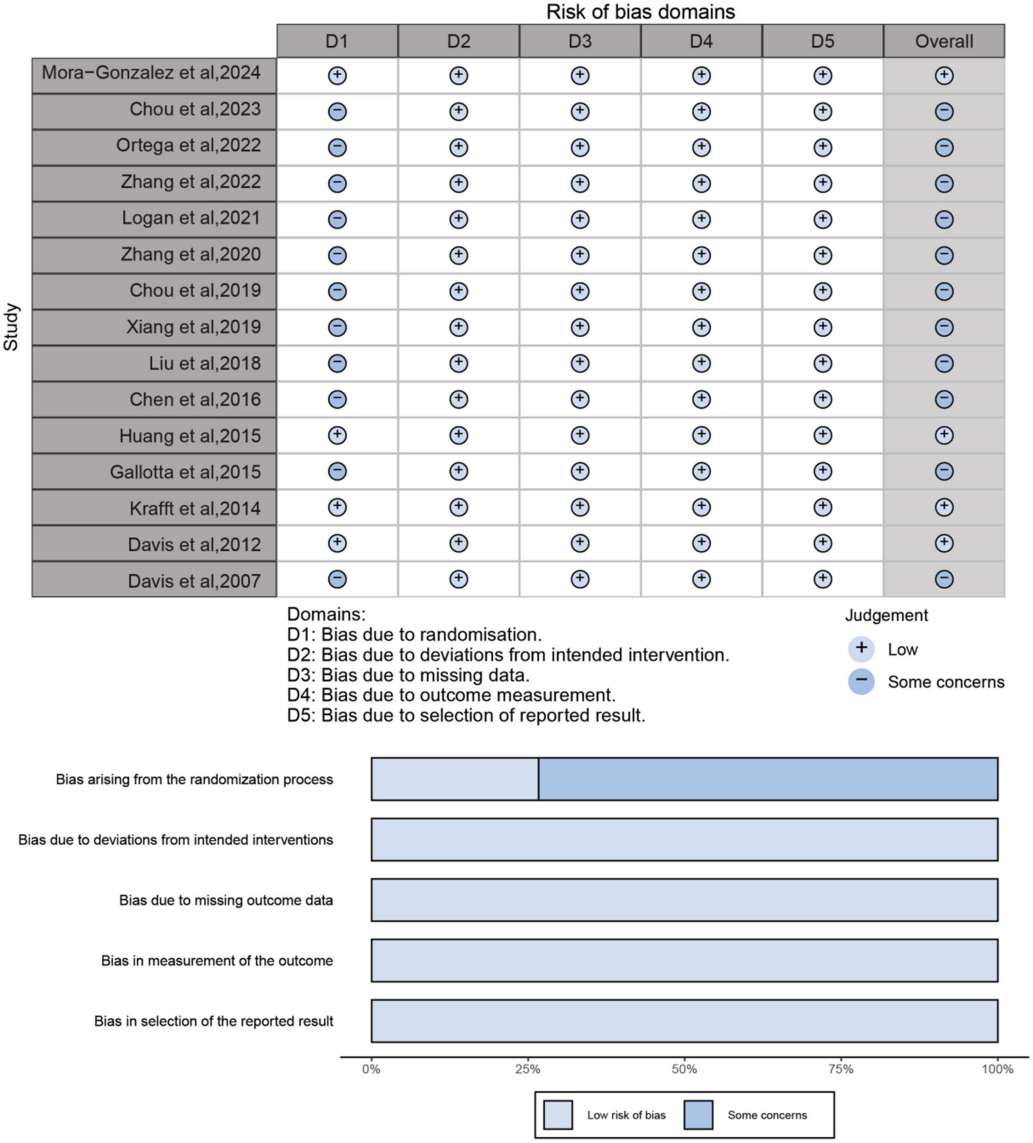
Risk of bias assessment in included studies.

The methodological quality of the included RCTs was assessed using the Physical Activity and Disability Research Centre Scale (PEDro) and Cochrane RoB2, respectively. Overall, the included studies showed moderate to good methodological quality, with a mean PEDro score of 6.8 (range: 4–8) (Table S1). Among the 15 RCTs, 12 were classified as good quality (total score: 6–8), and three were considered of poor quality (score ≤ 5). The risk of bias assessment based on the Cochrane RoB2 is summarized in Figure 2. Four of the 15 RCTs were judged to have an overall low risk of bias, while the remaining 11 raised some concerns, mainly due to inadequacies in the randomization process (e.g., unclear allocation concealment).

## Results of the meta-analysis

### Executive function

#### Overall effects of exercise on executive function

**Figure 3:**
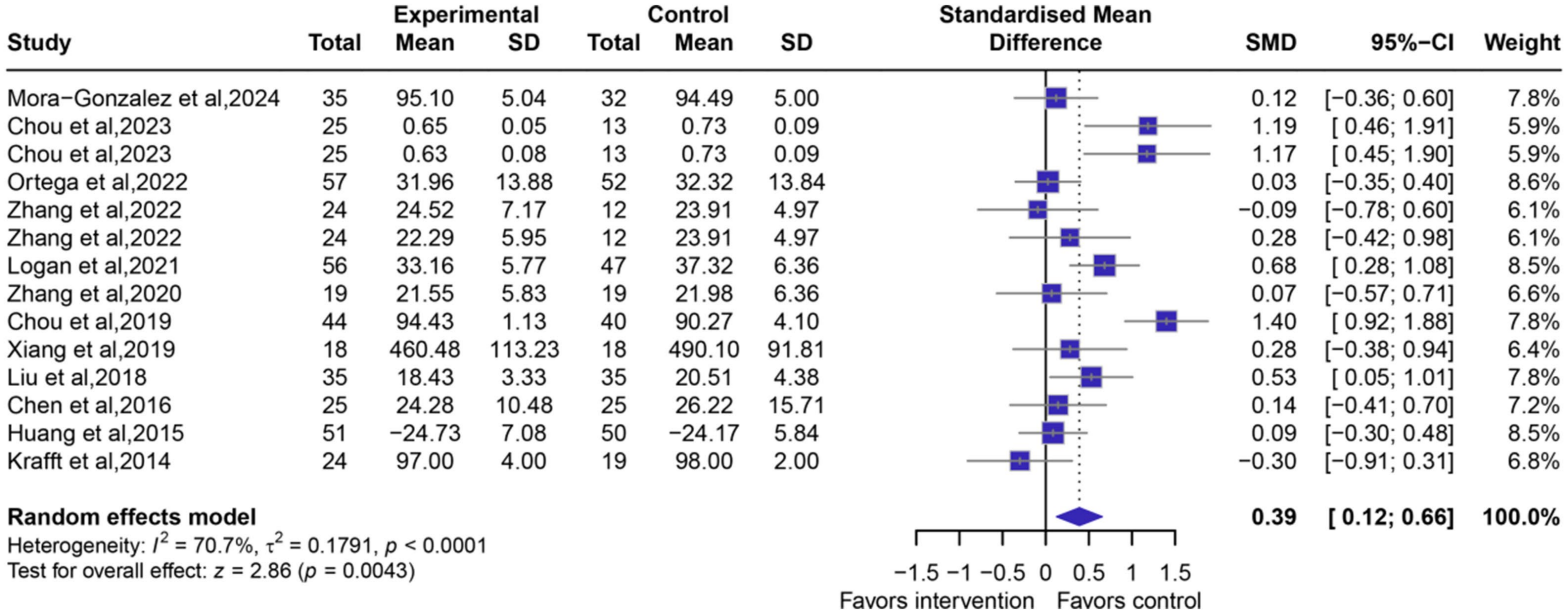
Forest plots of exercise intervention on executive function. A positive effect value indicates a improvement in executive function. The effect size is Hedges’g.

Fourteen intervention arms from 12 RCTs (*n =* 849 participants) were performed to assess the effects of exercise on executive function. The results indicated a significant beneficial effect of exercise interventions (*g*: 0.39, 95% CI: 0.12 to 0.66, *p* = 0.0043, Figure 3), with high levels of heterogeneity (*I^2^* = 70.7%) and no significant publication bias (*p* = 0.7032, Supplementary Figure S10). Sensitivity analysis demonstrated consistent effect sizes (SMD range: 0.30 to 0.44) with all 95% CI excluding the null value (all *p* < 0.05), and confirmed the robustness of the findings against both publication bias and individual study influences (Supplementary Figure S11A). In addition, exercise intervention significantly improved CAS-Assessed (the Cognitive Assessment System) executive function compared to control conditions (*g*: 0.19, 95% CI: 0.07 to 0.32, *p* = 0.0028, Supplementary Figure S12). There was no significant difference in effect sizes between dimensions (*p* > 0.05). Supplementary Figure S11D shows no significant publication bias (*p* = 0.2314).

Subgroup analyses identified significant modifiers of exercise efficacy on executive function, including intervention parameters (type/frequency/intensity/duration), age and BMI status. Aerobic exercise demonstrated a significantly greater improvement than multicomponent exercise (*g* = 0.47, 95% CI: 0.13 to 0.80, *p* = 0.0059 vs. *g* = 0.10, 95% CI: 0.17 to 0.37, *p* = 0.4736, Table 2; Supplementary Figure S1). Exercise interventions administered >3 times per week significantly improved executive function (*g* = 0.39, 95% CI: 0.05 to 0.73, *p* = 0.0257), whereas those administered ≤3 times per week did not achieve statistical significance (*g* = 0.39, 95% CI: −0.07 to 0.85, *p* = 0.0986, Table 2; Supplementary Figure S2). Moderate-to-vigorous exercise significantly enhanced executive function (*g* = 0.50, 95% CI: 0.10 to 0.89, *p* = 0.0132), while neither moderate-only nor vigorous-only intensity demonstrated statistically significant improvements (both *p* > 0.05, Table 2; Supplementary Figure S3). Exercise sessions lasting >30 min significantly improved executive function (*g* = 0.41, 95% CI: 0.08 to 0.73, *p* = 0.0139), while sessions ≤30 min did not achieve statistical significance (*g* = 0.35, 95% CI: −0.19 to 0.88, *p* = 0.2085, Table 2; Supplementary Figure S4). Weekly exercise volume >120 min significantly improved executive function (*g* = 0.28, 95% CI: 0.04 to 0.53, *p* = 0.0236), whereas volumes ≤120 min did not reach statistical significance (*g* = 0.58, 95% CI: −0.02 to 1.19, *p* = 0.0595, Table 2; Supplementary Figure S5). Long-term interventions (≥10 weeks) significantly improved executive function (*g* = 0.41, 95% CI: 0.07 to 0.75, *p* = 0.0184), whereas short-term programs (<10 weeks) did not achieve statistical significance (g = 0.36, 95% CI: −0.11 to 0.82, *p* = 0.1308, Table 2; Supplementary Figure S6). Both children (*g* = 0.44, 95% CI: 0.07 to 0.81, *p* = 0.0186) and adolescents (*g* = 0.29, 95% CI: 0.01 to 0.57, *p* = 0.0448) showed significant executive function improvements, though adolescents demonstrated a larger standardized mean difference (Table 2; Supplementary Figure S7). For BMI subgroups, overweight youth exhibited substantially greater improvements (*g* = 0.87, 95% CI: 0.37 to 1.36, *p* = 0.0006) than obesity (*g* = 0.13, 95% CI: −0.05 to 0.31, *p* = 0.1696, Table 2; Supplementary Figure S8). Meta-regression exploring heterogeneity sources revealed no significant effects of exercise dose parameters (duration/volume/frequency/total time) on executive function (*p* > 0.05), with consistent but non-significant negative associations with SMD (Supplementary Figure S9).

**Table 2:** Subgroup analysis of the effects of different moderating variables on executive function.

Moderator variable	Stratified subgroup	Numbers included in the study	SMD (95% CI)	I^2^ (%)	*p*
Exercise type	Aerobic exercise	11	0.47 [0.13,0.80]	74	0.0059
	Multicomponent exercise	3	0.10 [−0.17,0.37]	0	0.4736
Exercise frequency (sessions/week)	>3	8	0.39 [0.05,0.73]	65.6	0.0257
	≤3	6	0.39 [−0.07,0.85]	79	0.0986
Exercise intensity	Vigorous-only	2	0.09 [−0.40,0.59]	0	0.7085
	Moderate-to-vigorous	9	0.50 [0.10,0.89]	80.1	0.0132
	Moderate-only	3	0.29 [−0.02,0.61]	0	0.0683
Exercise duration (mins)	> 30	10	0.41 [0.08,0.73]	75.7	0.0139
	≤ 30	4	0.35 [−0.19,0.88]	58.7	0.2085
Total exercise time (mins/ per week)	> 120	9	0.28 [0.04,0.53]	55	0.0236
	≤ 120	5	0.58 [−0.02,1.19]	80	0.0595
Exercise period (weeks)	≥ 10	8	0.41 [0.07,0.75]	68.1	0.0184
	< 10	6	0.36 [−0.11,0.82]	77.7	0.1308
Age	Children	10	0.44 [0.07,0.81]	78.7	0.0186
	Adolescents	4	0.29 [0.01,0.57]	0	0.0448
BMI	Overweight	5	0.87 [0.37,1.36]	80.9	0.0006
	Obesity	9	0.13 [−0.05,0.31]	0	0.1696

#### Overall effects of exercise on attention function

**Figure 4:**
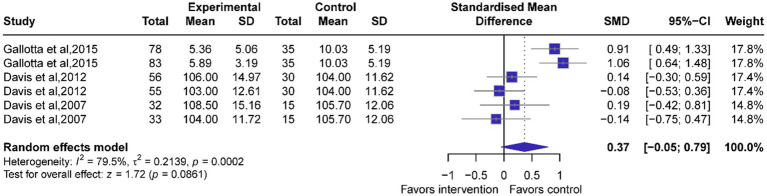
Forest plots of exercise intervention on attention function. A positive effect value indicates a improvement in attention function. The effect size is Hedges’g.

Six studies (*n =* 497 participants) were conducted to assess the effects of exercise on tests of attention function. Our results revealed no significant benefits over the control group despite a potential trend favoring the intervention (*g*:0.37, 95% CI: −0.05, 0.79, *p* = 0.0861, Figure 4), with a high level of heterogeneity (*I*^2^ = 79.5%) and no significant publication bias (*p* = 0.2507). Sensitivity analyses confirmed the benefits in all cases (Supplementary Figure S11B).

Subgroup analyses identified significant modifiers of exercise efficacy on executive function, including intervention parameters (frequency/intensity/duration). Compared to ≥5 sessions/week, interventions with <5 sessions/week demonstrated significantly greater improvement in attention (*g* = 0.98, 95% CI: 0.69–1.28; *p* < 0.0001, Table 3; Supplementary Figure S13). Sessions lasting ≥40 min demonstrated moderate attention improvement compared to <40-min sessions (*g* = 0.60, 95% CI: 0.13–1.07, *p* = 0.0124, Table 3; Supplementary Figure S14). Weekly exercise volumes ≥120 min demonstrated moderate attention improvement compared to <120-min volumes (*g* = 0.60, 95% CI: 0.13–1.07, *p* = 0.012, Table 3; Supplementary Figure S15). Exercise duration >15 weeks demonstrated significantly greater attention improvement than ≤15-week programs (*g* = 0.98, 95% CI: 0.69–1.28, *p* < 0.0001, Table 3; Supplementary Figure S16). Collectively, these findings indicate that reduced exercise frequency (<5 sessions/week), longer single-session duration (≥40 min), accumulated weekly exercise volume (≥120 min), and prolonged intervention (>15 weeks) are robustly associated with improved attentional performance. Meta-regression analysis revealed significant negative associations between attentional improvement and both intervention period (*β* = −0.2026, *p* < 0.0001) and increased session duration (*β* = −0.0228, *p* = 0.0223), while demonstrating positive correlations with weekly exercise volume and exercise frequency (*β* = 0.3376, *p* < 0.0001, Supplementary Figure S17).

**Table 3:** Subgroup analysis of the effects of different moderating variables on attention function.

Moderator variable	Stratified subgroup	Numbers included in the study	SMD (95%CI)	I^2^ (%)	*p*
Exercise frequency (sessions/week)	≥5	4	0.03 [−0.23,0.28]	0	0.8234
	<5	2	0.98 [0.69,1.28]	0	<0.0001
Exercise duration (mins)	≥40	4	0.60 [0.13,1.07]	75.6	0.0123
	<40	2	−0.10 [−0.46,0.26]	0	0.5793
Total exercise time (mins/ per week)	≥120	4	0.60 [0.13,1.07]	75.6	0.0123
	<120	2	−0.10 [−0.46,0.26]	0	0.5793
Exercise period (weeks)	>15	2	0.98 [0.69,1.28]	0	0.0001
	≤15	4	0.03 [−0.23,0.28]	0	0.8234

#### Overall effects of exercise on memory

**Figure 5:**
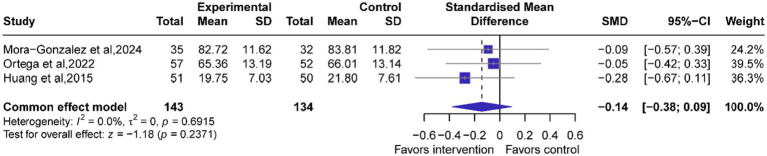
Forest plots of exercise intervention on memory function. A negative effect value indicates a improvement in memory function. The effect size is Hedges’g.

A total of 7 studies (*n =* 277 participants) assess the effects of exercise on memory function in children and adolescents. Our pooled analysis revealed no significant benefits of the intervention compared with the control group (*g*: −0.14 95% CI: −0.38, 0.09, *p* = 0.2371, Figure 5). There was no evidence of heterogeneity (*I*^2^ = 0%) and no significant publication bias (*p* = 0.9293) (Table 4).

**Table 4:** Characteristics of the included studies.

Author	*N*	Mean age	Mean BMI	Country	Comparison group	Exercise type	Exercise duration (wk)	Exercise frequency	Exercise volume (min/session)	Exercise intensity	Outcomes (measured)
Mora-Gonzalez et al., 2024 (45)	67	10	26.58	Spain	Wait-list	AE_RE	20	4	90	Moderate-to-vigorous (mixed)	EF: MFT Working memory: DNMS
Chou et al., 2023 (46)	38	11.23	20.76	China	Control	AE: CEMGs	10	5	40	Moderate-to-vigorous (mixed)	EF: Stroop test
Chou et al., 2023 (46)	38	11.23	20.76	China	Control	AE: CEMGs	10	5	20	Moderate-to-vigorous (mixed)	EF: Stroop test
Ortega et al., 2022 (47)	109	10	26.8	Spain	Control	Multicomponent_exercise	20	3	90	Moderate-to-vigorous (mixed)	EF: Stroop Color Word Test Working memory: DNMS
Zhang et al., 2022 (48)	36	11.56	26.02	China	Control	AE: Treadmill interval exercise	1	1	30	Vigorous only	EF: Stroop test
Zhang et al., 2022 (48)	36	11.56	26.02	China	Control	AE: Rope skipping (continuous)	1	1	30	Vigorous only	EF: Stroop test
Logan et al., 2021 (49)	103	8–10	25.34	USA	Wait-list	AE	39	5	120	Moderate-to-vigorous (mixed)	EF: Eriksen flanker task
Zhang et al., 2020 (50)	38	14.63	E:27.75:28.06	China	Control	AE: jump rope	1	1	20	Moderate only	EF: Stroop Color Word Test
Chou et al., 2019 (51)	84	12.19	24.84	China	Control	AE: Movement Games	8	3	40	Moderate-to-vigorous (mixed)	EF: Stroop test Determination test
Xiang et al., 2019 (52)	36	E:12.50:13.28	E:29.57:29.09	China	Wait-list	Multicomponent_exercise	6	6	300	Moderate-to-vigorous (mixed)	EF: Stroop test
Liu et al., 2018 (53)	70	E:13.94:14.17	E:28.01:29.92	China	Wait-list	AE:jump rope	12	2	75	Moderate only	EF: Stroop test
Chen et al., 2016 (54)	50	E:12.64:12.84	E:27.90:29.98	China	Wait-list	AE	13	4	40	Moderate only	EF: WCST
Huang et al., 2015 (55)	115	12.0	E:25.2:24.5	Denmark	Control	AE	6	7	180	Moderate-to-vigorous (mixed)	EF: Stroop test Memory: RCFT
Gallotta et al., 2015 (59)	156	8–11	25.1–27.7	Italy	Control	AE: Traditional PA	20	2	60	Moderate-to-vigorous (mixed)	Attention:d2-R
Gallotta et al., 2015 (59)	156	8–11	25.1–27.7	Italy	Control	AE: Coordinative PA	20	2	60	Moderate-to-vigorous (mixed)	Attention:d2-R
Krafft et al., 2014 (56)	43	E: 9.7: 9.9	E:1.91:1.93	USA	Wait-list	AE	35	5	40	Moderate-to-vigorous (mixed)	EF: flanker task Antisaccade
Davis et al., 2012 (58)	171	9.3	26	USA	Control	AE	13	5	20	Moderate-to-vigorous (mixed)	CAS
Davis et al., 2012 (58)	171	9.3	26	USA	Control	AE	13	5	40	Moderate-to-vigorous (mixed)	CAS
Davis et al., 2007 (57)	94	9.2	25.8	USA	Control	AE	15	5	20	Moderate-to-vigorous (mixed)	CAS
Davis et al., 2007 (57)	94	9.2	25.8	USA	Control	AE	15	5	40	Moderate-to-vigorous (mixed)	CAS

CEMGs, cognitively engaging movement games; MFT, modified flanker task; EF, Executive function; DNMS, Delayed non-matched-to-sample task; WCST, Wisconsin Card Sorting Test; RCFT, Rey Complex Figure Test; CAS, Cognitive Assessment System.

## Discussion

This systematic review and meta-analysis comprehensively evaluate the effects of different exercise variables on cognitive sub-domains in overweight or obesity children and adolescents. The results demonstrated that exercise interventions in this population significantly improved cognitive function, particularly executive functioning. Importantly, our analysis identified a potentially effective exercise prescription for executive function: aerobic moderate-to-vigorous intensity exercise at >3 sessions/week, >30 min/session, >120 min/week sustained for ≥10 weeks. Furthermore, attention was maximized under distinct parameters: <5 sessions/week, ≥40 min/session, ≥120 min/week, and >15-week intervention periods. These findings provide theoretical and practical evidence aimed at guiding community and clinical exercise prescribing to improve cognitive ability (especially the executive function and attention function) in overweight or obesity children and adolescents.

The present meta-analysis demonstrates that exercise interventions significantly enhance executive function in children and adolescents with overweight or obesity, encompassing both global and CAS-assessed domains. These findings are consistent with cross-age and cross-health-status evidence demonstrating that exercise confers universal benefits on executive function in healthy children, adults, older adults (60–65). The improvement observed in this study exhibits a potential dose–response relationship: sessions exceeding 30 min, accumulating to more than 120 min per week and maintaining for at least 10 weeks, may be the optimal pattern. These findings are consistent with previous reviews indicating that 25–40 min of moderate-to-vigorous exercise per session improves executive function in this population (32, 66, 67). Importantly, our study also shows that interventions involving more than three sessions per week for 10–12 weeks are most effective in improving cognitive function. This finding indicates that a 10- to 12-week exercise intervention cycle may represent a critical time interval for enhancing executive functioning in overweight or obese children and adolescents. Notably, this conclusion accords with the World Health Organization’s physical activity recommendations for children (68). Collectively, these findings indicate that modifying exercise-related variables may improve executive function in children and adolescents with overweight or obesity.

In children and adolescents with overweight or obesity, exercise appears to enhance executive function through multiple neural mechanisms. For example, increased functional connectivity between the anterior hippocampus and prefrontal cortex, as well as between the posterior cingulate cortex and networks including the executive control network, basal ganglia, and default mode network, has been linked to improvements in inhibitory control, working memory, and cognitive flexibility (69–71). Behaviorally, individuals who continuously exercise exhibit greater task-related brain activation, faster response times, and higher accuracy on incongruent trials (72). Neuroimaging evidence further indicates that exercise elevates prefrontal cortex activity while attenuating posterior parietal activation, suggesting that reorganization of functional brain patterns may underlie these cognitive benefits (73). Long-term exercise also enhances executive function through increased cortical activation and cerebral blood flow, facilitating attention, inhibitory control, and information processing (74). At the molecular level, chronic exercise elevates levels of brain-derived neurotrophic factor (BDNF) and other growth factors, promoting synaptogenesis, neuronal survival, and structural and functional plasticity within prefrontal and hippocampal circuits (75). Consistent with this, exercise interventions in youth have been shown to increase circulating BDNF, implying enhanced synaptic formation and neural adaptability (76). Collectively, these neural, hemodynamic, and molecular adaptations likely contribute to the observed improvements in executive function. Exercise programs lasting ≥10 weeks may be particularly aligned with BDNF-mediated plasticity in key cortical and subcortical regions. Future studies should employ longitudinal and interventional designs integrating multimodal neuroimaging (e.g., fMRI) with molecular and biochemical measures (e.g., serum or cerebrospinal fluid BDNF), and complementary preclinical models, to elucidate underlying mechanisms and establish causal links.

Subgroup analyses indicated a tendency that suggests the potential efficacy of exercise interventions in enhancing memory function in children and adolescents who are overweight or obesity. This finding is consistent with the conclusions drawn from previous studies (32, 66). However, some research reports have not found significant positive effects of exercise. This is because the types of intervention are different, or because there are not many studies available (19). Similar to several previous research reports, our meta-analysis also shows that exercise has no significant impact on attention (19, 30, 32, 66). In contrast, exercise has been shown to significantly enhance attention in children and adolescents with ADHD (19), suggesting that disease status or underlying metabolic characteristics may modulate cognitive responsiveness. For example, obesity-induced metabolic disorders may result in a physiological milieu that is less responsive and/or slower to adapt to external stimuli than that of metabolically healthy peers (3, 77). Additionally, our partial results indicated that exercise exerted stronger effects on executive function in overweight children than in those with obesity, suggesting differential sensitivity across cognitive domains. Methodological factors, such as small sample sizes, variation in intervention type and duration, and limited sensitivity of assessment tools, may also have contributed to the non-significant findings. Therefore, the absence of significant improvements on attention and memory may reflect both genuine neurocognitive differences and methodological constraints. Future studies should employ larger, well-controlled samples and consider stratification by metabolic or disease status to more precisely delineate the cognitive effects of exercise.

### Limitations and implications

This study strictly adhered to the guidelines outlined in the PRISMA statement. However, 5 limitations should be considered in future research: 1) Although the literature search was rigorously conducted according to PRISMA standards, unpublished works may have been overlooked; 2) Current exercise trials predominantly focus on aerobic exercise, with limited comparison to other modalities, thus future research should expand the scope of exercise types for comparative analysis; 3) Exercise variables have primarily been defined by executive function, and future research and reviews should also address the limitations associated with overall cognitive function improvement, including exercise duration, timing, frequency, and intensity; 4) Although a dose–response relationship between physical activity and cognitive improvement was observed, this finding should be interpreted with caution due to the limited number of trials involving overweight or obesity children and adolescents. Future research requires more standardized, high-quality randomized controlled trials with larger sample sizes and rigorous measurement of activity dose and cognitive indicators to confirm these potential effects; 5) Finally, the lack of multidimensional cognitive measurement indicators represents another limitation, as the current focus has been mainly on executive function with fewer assessments of other cognitive metrics; additionally, the absence of neuroimaging evidence and molecular biology indicators may reduce the reliability of clinical research conclusions; 6) It is worth noting the limits of physical activities for obesity children and general or partial contraindications. Prolonged activities such as running or jumping may increase the risk of skeletal injury and joint stress in this population. In addition, hypertension, a common comorbidity among individuals with overweight or obesity, can be exacerbated by high-intensity exercise. Therefore, investigating the personalized and effective exercise intervention strategies underlying these improvements represents a valuable direction for future work.

From a practical perspective, healthcare and education researchers can prioritize the integration of moderate-to-vigorous physical activity into intervention strategies to optimize cognitive health among children and adolescents with overweight or obesity. Additionally, public health policymakers could leverage these findings to develop scalable, community-based programs (such as after-school sports initiatives) to promote cognitive and physical wellbeing, particularly in regions with a high prevalence of overweight and obesity.

## Conclusion

Our systematic review and meta-analysis suggest that exercise interventions in overweight or obesity children and adolescents may have beneficial effects on cognitive performance. Especially for executive function, we found that moderate-intensity aerobic exercise performed >3 times/week, >30 min/session, for ≥10 weeks significantly improved executive function in overweight or obesity children and adolescents. Furthermore, attention was maximized under distinct parameters: <5 sessions/week, ≥40 min/session, ≥120 min/week, and >15-week intervention periods. Further high-quality RCTs with larger sample sizes are, however, needed to strengthen the evidence and to explore the potential moderating effect of intervention and participant characteristics.

## Data Availability

The original contributions presented in the study are included in the article/Supplementary material, further inquiries can be directed to the corresponding authors.

## References

[1] Sweatt K Garvey WT Martins C. Strengths and limitations of BMI in the diagnosis of obesity: what is the path forward? Curr Obes Rep. (2024) 13:584–95. doi: 10.1007/s13679-024-00580-1, 38958869 PMC11306271

[2] Blüher M. Obesity: global epidemiology and pathogenesis. Nat Rev Endocrinol. (2019) 15:288–98. doi: 10.1038/s41574-019-0176-8, 30814686

[3] Sun Q Wang J Wang H Yu H Wan K Ma F . Effect of long-term taurine supplementation on the lipid and glycaemic profile in adults with overweight or obesity: a systematic review and meta-analysis. Nutrients. (2024) 17:55. doi: 10.3390/nu17010055, 39796489 PMC11722866

[4] Ng M Fleming T Robinson M Thomson B Graetz N Margono C . Global, regional, and national prevalence of overweight and obesity in children and adults during 1980–2013: a systematic analysis for the global burden of disease study 2013. Lancet. (2014) 384:766–81. doi: 10.1016/S0140-6736(14)60460-8, 24880830 PMC4624264

[5] Abarca-Gómez L Abdeen ZA Hamid ZA Abu-Rmeileh NM Acosta-Cazares B Acuin C . Worldwide trends in body-mass index, underweight, overweight, and obesity from 1975 to 2016: a pooled analysis of 2416 population-based measurement studies in 128·9 million children, adolescents, and adults. Lancet. (2017) 390:2627–42. doi: 10.1016/S0140-6736(17)32129-3, 29029897 PMC5735219

[6] Zhang X Liu J Ni Y Yi C Fang Y Ning Q . Global prevalence of overweight and obesity in children and adolescents. JAMA Pediatr. (2024) 178:800–13. doi: 10.1001/jamapediatrics.2024.1576, 38856986 PMC11165417

[7] Homepage | World Obesity Day. Available online at: https://www.worldobesityday.org/

[8] Sharma V Coleman S Nixon J Sharples L Hamilton-Shield J Rutter H . A systematic review and meta-analysis estimating the population prevalence of comorbidities in children and adolescents aged 5 to 18 years. Obes Rev. (2019) 20:1341–9. doi: 10.1111/obr.12904, 31342672 PMC6851579

[9] Dye L Boyle NB Champ C Lawton C. The relationship between obesity and cognitive health and decline. Proc Nutr Soc. (2017) 76:443–54. doi: 10.1017/S0029665117002014, 28889822

[10] Oliveras-Cañellas N Castells-Nobau A de la Vega-Correa L Latorre-Luque J Motger-Albertí A Arnoriaga-Rodriguez M . Adipose tissue coregulates cognitive function. Sci Adv. (2023) 9:eadg4017. doi: 10.1126/sciadv.adg4017, 37566655 PMC10421051

[11] Morys F Yu E Shishikura M Paquola C Vainik U Nave G . Neuroanatomical correlates of genetic risk for obesity in children. Transl Psychiatry. (2023) 13:1–12. doi: 10.1038/s41398-022-02301-5, 36596778 PMC9810659

[12] Saeed S Bonnefond A Froguel P. Obesity: exploring its connection to brain function through genetic and genomic perspectives. Mol Psychiatry. (2025) 30:651–8. doi: 10.1038/s41380-024-02737-9, 39237720 PMC11746128

[13] Steptoe A Frank P. Obesity and psychological distress. Philos Trans R Soc Lond Ser B Biol Sci. (2023) 378:20220225. doi: 10.1098/rstb.2022.0225, 37661745 PMC10475872

[14] Wardle J Cooke L. The impact of obesity on psychological well-being. Best Pract Res Clin Endocrinol Metab. (2005) 19:421–40. doi: 10.1016/j.beem.2005.04.006, 16150384

[15] Chen A Guo C Qu S. The effect of exercise intervention on inhibitory function in obese and overweight children and adolescents: a systematic review and meta-analysis. BMC Pediatr. (2025) 25:17. doi: 10.1186/s12887-024-05362-1, 39789470 PMC11715291

[16] Wang J Yang Y Li L Yang X Guo X Yuan X . Comparative efficacy of physical activity types on executive functions in children and adolescents: a network meta-analysis of randomized controlled trials. J Sci Med Sport. (2024) 27:187–96. doi: 10.1016/j.jsams.2023.11.006, 38042755

[17] Dastamooz S Sadeghi-Bahmani D Farahani MHD Wong SHS Yam JCS Tham CCY . The efficacy of physical exercise interventions on mental health, cognitive function, and ADHD symptoms in children and adolescents with ADHD: an umbrella review. eClinicalMedicine. (2023) 62:102137. doi: 10.1016/j.eclinm.2023.102137, 37599910 PMC10432969

[18] Liang X Li R Wong SHS Sum RKW Wang P Yang B . The effects of exercise interventions on executive functions in children and adolescents with autism Spectrum disorder: a systematic review and Meta-analysis. Sports Med. (2022) 52:75–88. doi: 10.1007/s40279-021-01545-3, 34468951

[19] Martin A Booth JN Laird Y Sproule J Reilly JJ Saunders DH. Physical activity, diet and other behavioural interventions for improving cognition and school achievement in children and adolescents with obesity or overweight. Cochrane Database Syst Rev. (2018) 2018:CD009728. doi: 10.1002/14651858.CD009728.pub4, 29376563 PMC6491168

[20] Liu C Liang X Sit CHP. Physical activity and mental health in children and adolescents with neurodevelopmental disorders. JAMA Pediatr. (2024) 178:247–57. doi: 10.1001/jamapediatrics.2023.6251, 38285440 PMC10825789

[21] O’ConnorEA EvansCV HenningerM RedmondN SengerCA. Interventions for weight management in children and adolescents. JAMA. (2024) 332:233. doi: 10.1001/jama.2024.6739, 38888913

[22] Fang Y Ma Y Mo D Zhang S Xiang M Zhang Z. Methodology of an exercise intervention program using social incentives and gamification for obese children. BMC Public Health. (2019) 19:686. doi: 10.1186/s12889-019-6992-x, 31159776 PMC6547593

[23] Quiroga R Nistal E Estébanez B Porras D Juárez-Fernández M Martínez-Flórez S . Exercise training modulates the gut microbiota profile and impairs inflammatory signaling pathways in obese children. Exp Mol Med. (2020) 52:1048–61. doi: 10.1038/s12276-020-0459-0, 32624568 PMC8080668

[24] Wang P Meng Y Tong J Jiang T. Effects of exercise intervention on executive function in children with overweight and obesity: a systematic review and meta-analysis. PeerJ. (2025) 13:e19273. doi: 10.7717/peerj.19273, 40247830 PMC12005193

[25] Lin C Li D Wang X Yang S. Chronic exercise interventions for executive function in overweight children: a systematic review and meta-analysis. Front. Sports Act. Living. (2024) 6:1336648. doi: 10.3389/fspor.2024.1336648, 38435336 PMC10907994

[26] Chen A Guo C Qu S. The effect of exercise intervention on inhibitory function in obese and overweight children and adolescents: a systematic review and meta-analysis. BMC Pediatr. (2025) 25:17. doi: 10.1186/s12887-024-05362-1, 39789470 PMC11715291

[27] Wang Y Wang H Zhao H. Effects of aerobic exercise on executive function among overweight and obese children: a systematic review and meta-analysis. Front Psychol. (2024) 15:1485610. doi: 10.3389/fpsyg.2024.1485610, 39529725 PMC11551034

[28] Sun X Li Y Cai L Wang Y. Effects of physical activity interventions on cognitive performance of overweight or obese children and adolescents: a systematic review and meta-analysis. Pediatr Res. (2021) 89:46–53. doi: 10.1038/s41390-020-0941-3, 32388536

[29] Wang P Meng Y Tong J Jiang T. Effects of exercise intervention on executive function in children with overweight and obesity: a systematic review and meta-analysis. PeerJ. (2025) 13:e19273. doi: 10.7717/peerj.19273, 40247830 PMC12005193

[30] Lin C Li D Wang X Yang S. Chronic exercise interventions for executive function in overweight children: a systematic review and meta-analysis. Front Sports Act Living. (2024) 6:1336648. doi: 10.3389/fspor.2024.1336648, 38435336 PMC10907994

[31] Chen A Guo C Qu S. The effect of exercise intervention on inhibitory function in obese and overweight children and adolescents: a systematic review and meta-analysis. BMC Pediatr. (2025) 25:17. doi: 10.1186/s12887-024-05362-1, 39789470 PMC11715291

[32] Wang Y Wang H Zhao H. Effects of aerobic exercise on executive function among overweight and obese children: a systematic review and meta-analysis. Front Psychol. (2024) 15:1485610. doi: 10.3389/fpsyg.2024.1485610, 39529725 PMC11551034

[33] Sun X Li Y Cai L Wang Y. Effects of physical activity interventions on cognitive performance of overweight or obese children and adolescents: a systematic review and meta-analysis. Pediatr Res. (2021) 89:46–53. doi: 10.1038/s41390-020-0941-3, 32388536

[34] Cumpston M Li T Page MJ Chandler J Welch VA Higgins JP . Updated guidance for trusted systematic reviews: a new edition of the Cochrane handbook for systematic reviews of interventions. Cochrane Database Syst Rev. (2019) 10:ED000142. doi: 10.1002/14651858.ED000142, 31643080 PMC10284251

[35] Ogden CL Kuczmarski RJ Flegal KM Mei Z Guo S Wei R . Centers for Disease Control and Prevention 2000 growth charts for the United States: improvements to the 1977 National Center for Health Statistics version. Pediatrics. (2002) 109:45–60. doi: 10.1542/peds.109.1.45, 11773541

[36] Peters JL Sutton AJ Jones DR Abrams KR Rushton L. Contour-enhanced meta-analysis funnel plots help distinguish publication bias from other causes of asymmetry. J Clin Epidemiol. (2008) 61:991–6. doi: 10.1016/j.jclinepi.2007.11.010, 18538991

[37] Egger M Smith GD Schneider M Minder C. Bias in meta-analysis detected by a simple, graphical test. BMJ. (1997) 315:629–34. doi: 10.1136/bmj.315.7109.629, 9310563 PMC2127453

[38] Viechtbauer W. Conducting meta-analyses in R with the metafor package. J Stat Softw. (2010) 36:1–48. doi: 10.18637/jss.v036.i03

[39] Meta-analysis with R. New York, NY: Springer.

[40] Veroniki AA Jackson D Viechtbauer W Bender R Bowden J Knapp G . Methods to estimate the between-study variance and its uncertainty in meta-analysis. Res Synth Methods. (2016) 7:55–79. doi: 10.1002/jrsm.1164, 26332144 PMC4950030

[41] Chang C-L Wu S-Y Ling DI Tsai C-L. The effect of exercise interventions on executive function in individuals with Parkinson’s disease: a meta-analysis of main and moderator effects. Biol Psychol. (2025) 199:109061. doi: 10.1016/j.biopsycho.2025.109061, 40482991

[42] Axon E Dwan K Richardson R. Multiarm studies and how to handle them in a meta-analysis: a tutorial. Cochrane Evid Synth Methods. (2023) 1:e12033. doi: 10.1002/cesm.12033, 40476010 PMC11795958

[43] Goodwin VA Abbott RA Whear R Bethel A Ukoumunne OC Thompson-Coon J . Multiple component interventions for preventing falls and fall-related injuries among older people: systematic review and meta-analysis. BMC Geriatr. (2014) 14:15. doi: 10.1186/1471-2318-14-15, 24495705 PMC3928080

[44] Higgins JPT. Measuring inconsistency in meta-analyses. BMJ. (2003) 327:557–60. doi: 10.1136/bmj.327.7414.557, 12958120 PMC192859

[45] Mora-Gonzalez J Esteban-Cornejo I Solis-Urra P Rodriguez-Ayllon M Cadenas-Sanchez C Hillman CH . The effects of an exercise intervention on neuroelectric activity and executive function in children with overweight/obesity: the ActiveBrains randomized controlled trial. Scand J Med Sci Sports. (2024) 34:e14486. doi: 10.1111/sms.14486, 37691352

[46] Chou C Kao S Pan C McCullick B Fu H Wang C. Cognitively engaging movement games improve interference control and academic performance in overweight children: a randomized control trial. Scand J Med Sci Sports. (2023) 33:521–34. doi: 10.1111/sms.14264, 36334308

[47] Ortega FB Mora-Gonzalez J Cadenas-Sanchez C Esteban-Cornejo I Migueles JH Solis-Urra P . Effects of an exercise program on brain health outcomes for children with overweight or obesity. JAMA Netw Open. (2022) 5:e2227893. doi: 10.1001/jamanetworkopen.2022.27893, 36040742 PMC9428743

[48] Zhang L Wang D Liu S Ren F-F Chi L Xie C. Effects of acute high-intensity interval exercise and high-intensity continuous exercise on inhibitory function of overweight and obese children. Int J Environ Res Public Health. (2022) 19:10401. doi: 10.3390/ijerph191610401, 36012036 PMC9408170

[49] LoganNE RaineLB DrolletteES CastelliDM KhanNA KramerAF . The differential relationship of an afterschool physical activity intervention on brain function and cognition in children with obesity and their normal weight peers. Pediatr Obes. (2021) 16:e12708. doi: 10.1111/ijpo.12708, 33249759

[50] Zhang L Chu C-H Liu J-H Chen F-T Nien J-T Zhou C . Acute coordinative exercise ameliorates general and food-cue related cognitive function in obese adolescents. J Sports Sci. (2020) 38:953–60. doi: 10.1080/02640414.2020.1737386, 32156187

[51] Chou C-C Chen K-C Huang M-Y Tu H-Y Huang C-J. Can movement games enhance executive function in overweight children? A randomized controlled trial. Cham: Springer (2019).

[52] Xiang M-Q Liao J-W Huang J-H Deng H-L Wang D Xu Z . Effect of a combined exercise and dietary intervention on self-control in obese adolescents. Front Psychol. (2019) 10:1385. doi: 10.3389/fpsyg.2019.01385, 31316417 PMC6610291

[53] Liu J-H Alderman BL Song T-F Chen F-T Hung T-M Chang Y-K. A randomized controlled trial of coordination exercise on cognitive function in obese adolescents. Psychol Sport Exerc. (2018) 34:29–38. doi: 10.1016/j.psychsport.2017.09.003 PMC7189777 32351324

[54] Chen S-R Tseng C-L Kuo S-Y Chang Y-K. Effects of a physical activity intervention on autonomic and executive functions in obese young adolescents: a randomized controlled trial. Health Psychol. (2016) 35:1120–5. doi: 10.1037/hea0000390, 27454114

[55] Huang T Larsen KT Jepsen JRM Møller NC Thorsen AK Mortensen EL . Effects of an obesity intervention program on cognitive function in children: a randomized controlled trial. Obesity. (2015) 23:2101–8. doi: 10.1002/oby.21209, 26337394

[56] Krafft CE Schwarz NF Chi L Weinberger AL Schaeffer DJ Pierce JE . An 8-month randomized controlled exercise trial alters brain activation during cognitive tasks in overweight children. Obesity. (2014) 22:232–42. doi: 10.1002/oby.20518, 23788510 PMC4077546

[57] Davis CL Tomporowski PD Boyle CA Waller JL Miller PH Naglieri JA . Effects of aerobic exercise on overweight children’s cognitive functioning. Res Q Exerc Sport. (2007) 78:510–9. doi: 10.1080/02701367.2007.10599450, 18274222 PMC2662758

[58] Davis CL Tomporowski PD McDowell JE Austin BP Miller PH Yanasak NE . Exercise improves executive function and achievement and alters brain activation in overweight children: a randomized, controlled trial. Health Psychol. (2011) 30:91–8. doi: 10.1037/a0021766, 21299297 PMC3057917

[59] Gallotta MC Emerenziani GP Iazzoni S Meucci M Baldari C Guidetti L. Impacts of coordinative training on normal weight and overweight/obese children’s attentional performance. Front Hum Neurosci. (2015) 9:577. doi: 10.3389/fnhum.2015.00577, 26578925 PMC4623610

[60] Zhang M Jia J Yang Y Zhang L Wang X. Effects of exercise interventions on cognitive functions in healthy populations: a systematic review and meta-analysis. Ageing Res Rev. (2023) 92:102116. doi: 10.1016/j.arr.2023.102116, 37924980

[61] Ye M Song T Xia H Hou Y Chen A. Effects of aerobic exercise on executive function of healthy middle-aged and older adults: a systematic review and meta-analysis. Int J Nurs Stud. (2024) 160:104912. doi: 10.1016/j.ijnurstu.2024.104912, 39326271

[62] Wang J Yang Y Li L Yang X Guo X Yuan X . Comparative efficacy of physical activity types on executive functions in children and adolescents: a network meta-analysis of randomized controlled trials. J Sci Med Sport. (2024) 27:187–96. doi: 10.1016/j.jsams.2023.11.006, 38042755

[63] Xue Y Yang Y Huang T. Effects of chronic exercise interventions on executive function among children and adolescents: a systematic review with meta-analysis. Br J Sports Med. (2019) 53:1397–404. doi: 10.1136/bjsports-2018-099825, 30737201

[64] Chen F-T Etnier JL Chan K-H Chiu P-K Hung T-M Chang Y-K. Effects of exercise training interventions on executive function in older adults: a systematic review and Meta-analysis. Sports Med. (2020) 50:1451–67. doi: 10.1007/s40279-020-01292-x, 32447717 PMC7376513

[65] Li D Miao C Wang D Li C. Effect of physical activity interventions on executive functions in school-age children with ADHD: a meta-analysis of randomized controlled trials. J Affect Disord. (2025) 378:175–90. doi: 10.1016/j.jad.2025.01.155, 40010649

[66] Wang P Meng Y Tong J Jiang T. Effects of exercise intervention on executive function in children with overweight and obesity: a systematic review and meta-analysis. PeerJ. (2025) 13:e19273. doi: 10.7717/peerj.19273, 40247830 PMC12005193

[67] Chen A Guo C Qu S. The effect of exercise intervention on inhibitory function in obese and overweight children and adolescents: a systematic review and meta-analysis. BMC Pediatr. (2025) 25:17. doi: 10.1186/s12887-024-05362-1, 39789470 PMC11715291

[68] WHO EMRO. What is the recommended amount of exercise? | Promoting physical activity | Physical activity | Health education and promotion. Available online at: https://www.emro.who.int/health-education/physical-activitiy/promoting-physical-activity/What-is-the-recommended-amount-of-exercise.html.

[69] Esteban-Cornejo I Stillman CM Rodriguez-Ayllon M Kramer AF Hillman CH Catena A . Physical fitness, hippocampal functional connectivity and academic performance in children with overweight/obesity: the ActiveBrains project. Brain Behav Immun. (2021) 91:284–95. doi: 10.1016/j.bbi.2020.10.006, 33049365

[70] Legget KT Wylie KP Cornier M-A Melanson EL Paschall CJ Tregellas JR. Exercise-related changes in between-network connectivity in overweight/obese adults. Physiol Behav. (2016) 158:60–7. doi: 10.1016/j.physbeh.2016.02.031, 26921099 PMC4803578

[71] Levakov G Kaplan A Yaskolka Meir A Rinott E Tsaban G Zelicha H . Neural correlates of future weight loss reveal a possible role for brain-gastric interactions. NeuroImage. (2021) 224:117403. doi: 10.1016/j.neuroimage.2020.117403, 32979521

[72] Wang M Wu S Ma Q Hu H Liu Y Wang Y . The role of exercise in restoring executive function: a comparison of tobacco-exposed college athletes and sedentary students. Front Physiol. (2024) 15:1499587. doi: 10.3389/fphys.2024.1499587, 39687517 PMC11646985

[73] Davis CL Tomporowski PD McDowell JE Austin BP Miller PH Yanasak NE . Exercise improves executive function and achievement and alters brain activation in overweight children: a randomized, controlled trial. Health Psychol. (2011) 30:91–8. doi: 10.1037/a0021766, 21299297 PMC3057917

[74] Chan Y-S Jang J-T Ho C-S. Effects of physical exercise on children with attention deficit hyperactivity disorder. Biom J. (2022) 45:265–70. doi: 10.1016/j.bj.2021.11.011, 34856393 PMC9250090

[75] Cotman C. Exercise: a behavioral intervention to enhance brain health and plasticity. Trends Neurosci. (2002) 25:295–301. doi: 10.1016/S0166-2236(02)02143-4, 12086747

[76] Shao X He L Liu Y. The effects of exercise interventions on brain-derived neurotrophic factor levels in children and adolescents: a meta-analysis. Neural Regen Res. (2025) 20:1513–20. doi: 10.4103/NRR.NRR-D-23-01296, 39075917 PMC11624860

[77] Zorena K Jachimowicz-Duda O Ślęzak D Robakowska M Mrugacz M. Adipokines and obesity. Potential link to metabolic disorders and chronic complications. Int J Mol Sci. (2020) 21:3570. doi: 10.3390/ijms21103570, 32443588 PMC7278967

