# Supplementary material for: Effects of exercise on cognitive function in children and adolescents with overweight or obesity: a systematic review and meta-analysis of randomized controlled trials
Source: Front Public Health. 2025 Dec 12;13:1694170. doi: 10.3389/fpubh.2025.1694170 (PMC12741061; doi:10.3389/fpubh.2025.1694170)
Supplement: Supplementary file 1 [file Data_Sheet_1.doc]

**Supplementary material**

Effects of exercise type on **executive function**

**Subgroup analysis by exercise type demonstrated that aerobic exercise significantly improved executive function (g: 0.47, 95% CI: 0.13 to 0.80, p=0.0059) compared to multicomponent exercise (g: 0.10, 95% CI: 0.17 to 0.37, p=0.4736) (Figure S1) .**


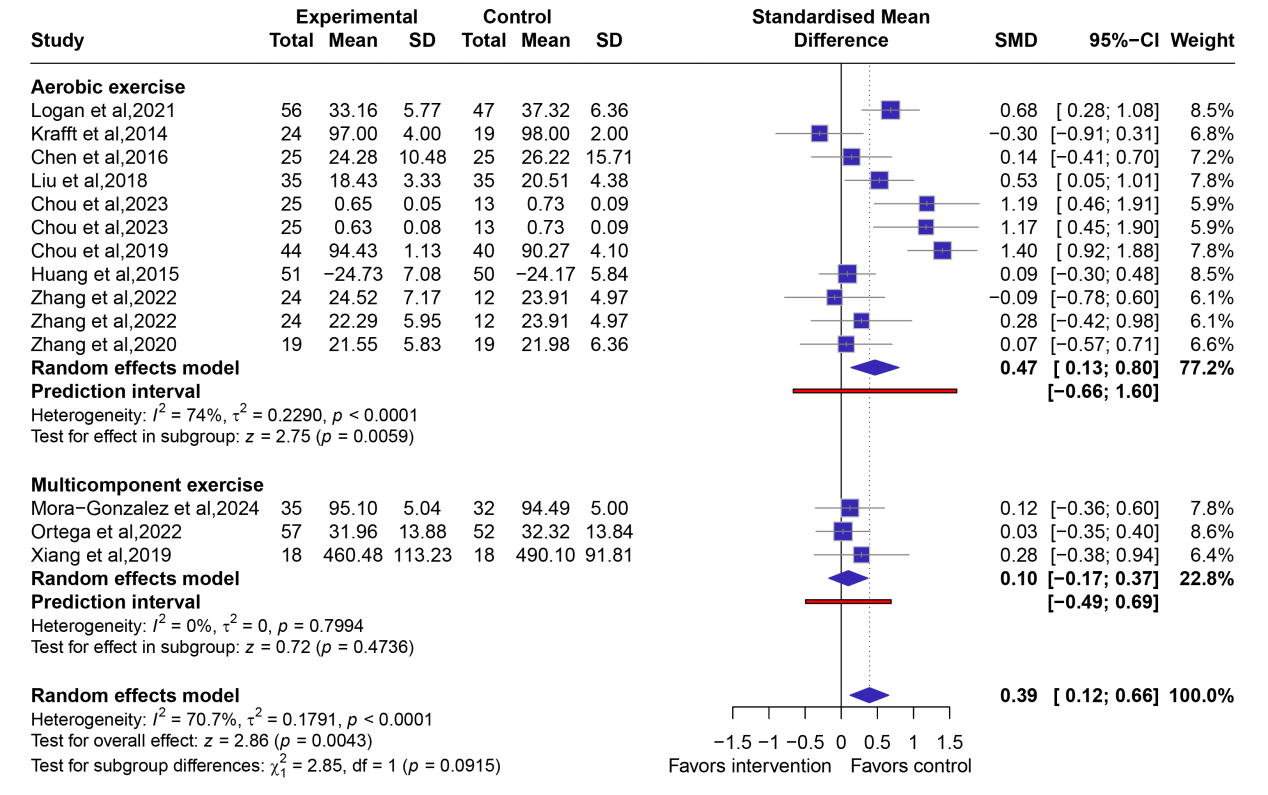


Figure S1. Subgroup analysis of exercise type on **executive function**.

A positive effect value indicates a improvement in executive function. The effect size is Hedges’g.

Effects of exercise frequency on **executive function**

Subgroup analysis stratified by frequency suggested that exercise interventions administered more than 3 times per week significantly improved executive function (g：0.39, 95% CI: 0.05, 0.73; p = 0.0257), whereas those conducted 3 times or fewer per week did not achieve statistical significance (g : 0.39, 95% CI: -0.07, 0.85; p = 0.0986), as illustrated in Figure S2.


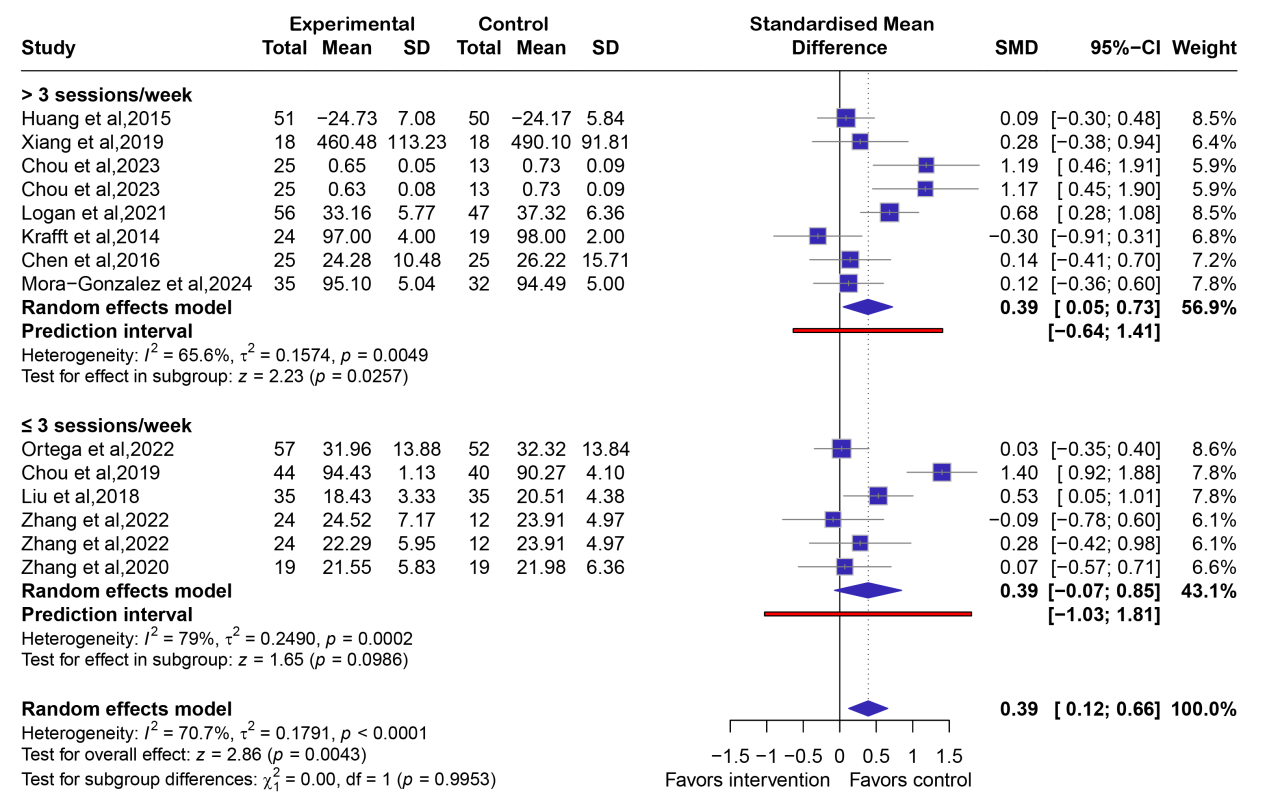


Figure S2. Subgroup analysis of exercise frequency on **executive function**.

A positive effect value indicates a improvement in executive function. The effect size is Hedges’g.

Effects of exercise intensity on **executive function**

Subgroup analysis stratified by exercise intensity suggested that moderate-to-vigorous exercise interventions significantly improved executive function (g : 0.50, 95% CI: 0.10, 0.89; p = 0.0132), whereas moderate-only and vigorous-only intensity levels were not significant( p > 0.05), as illustrated in Figure S3.

**
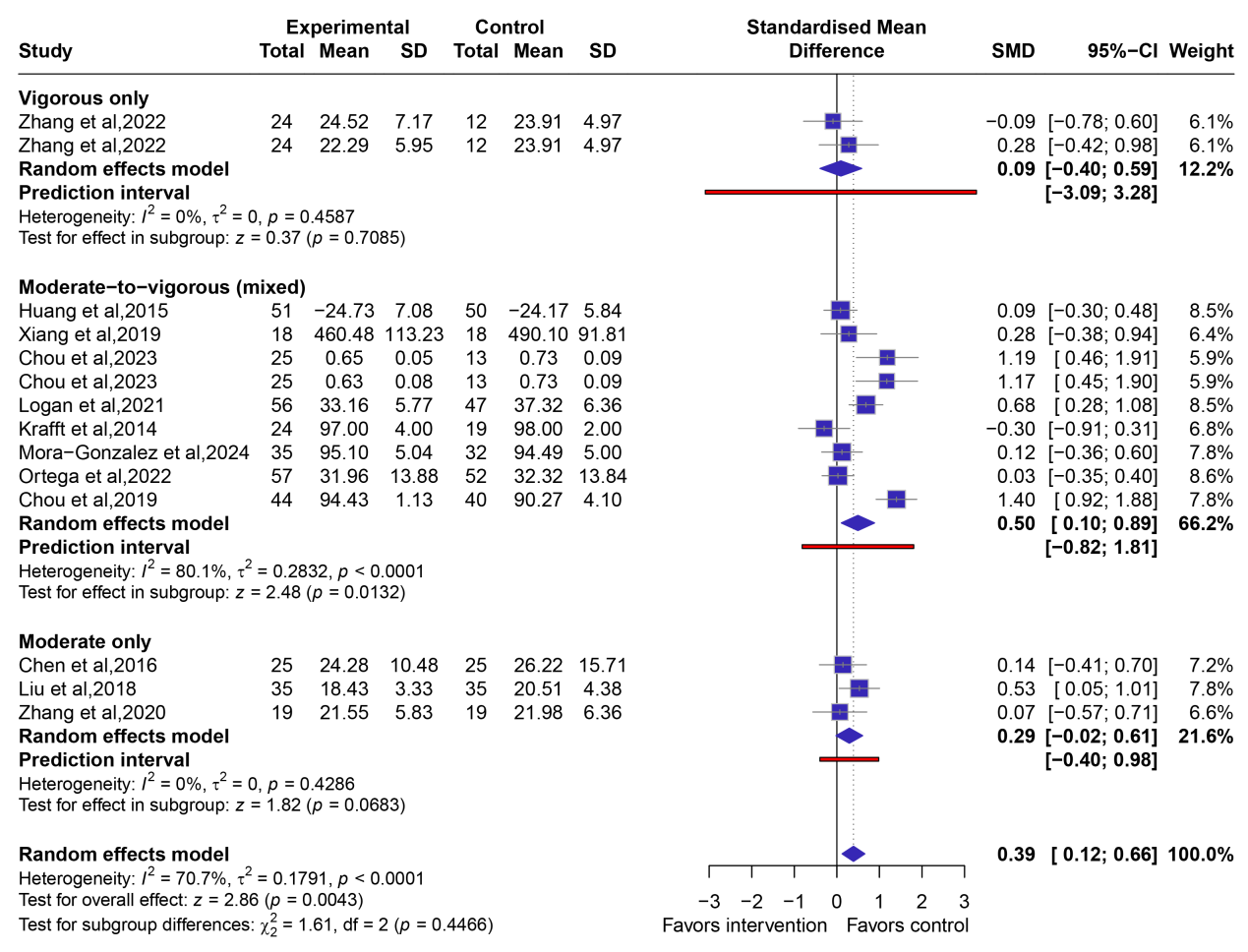
**

Figure S3. Subgroup analysis of exercise intensity on **executive function**.

A positive effect value indicates a improvement in executive function. The effect size is Hedges’g.

Effects of session duration on **executive function**

Subgroup analysis revealed that exercise sessions conducted more than 30 minutes per session significantly improved executive function (g : 0.41, 95% CI: 0.08, 0.73, p=0.0139), while those conducted 30 minutes or fewer per session did not reach statistical significance (g: 0.35, 95% CI: -0.19, 0.88, p=0.2085), as shown in Figure S4.


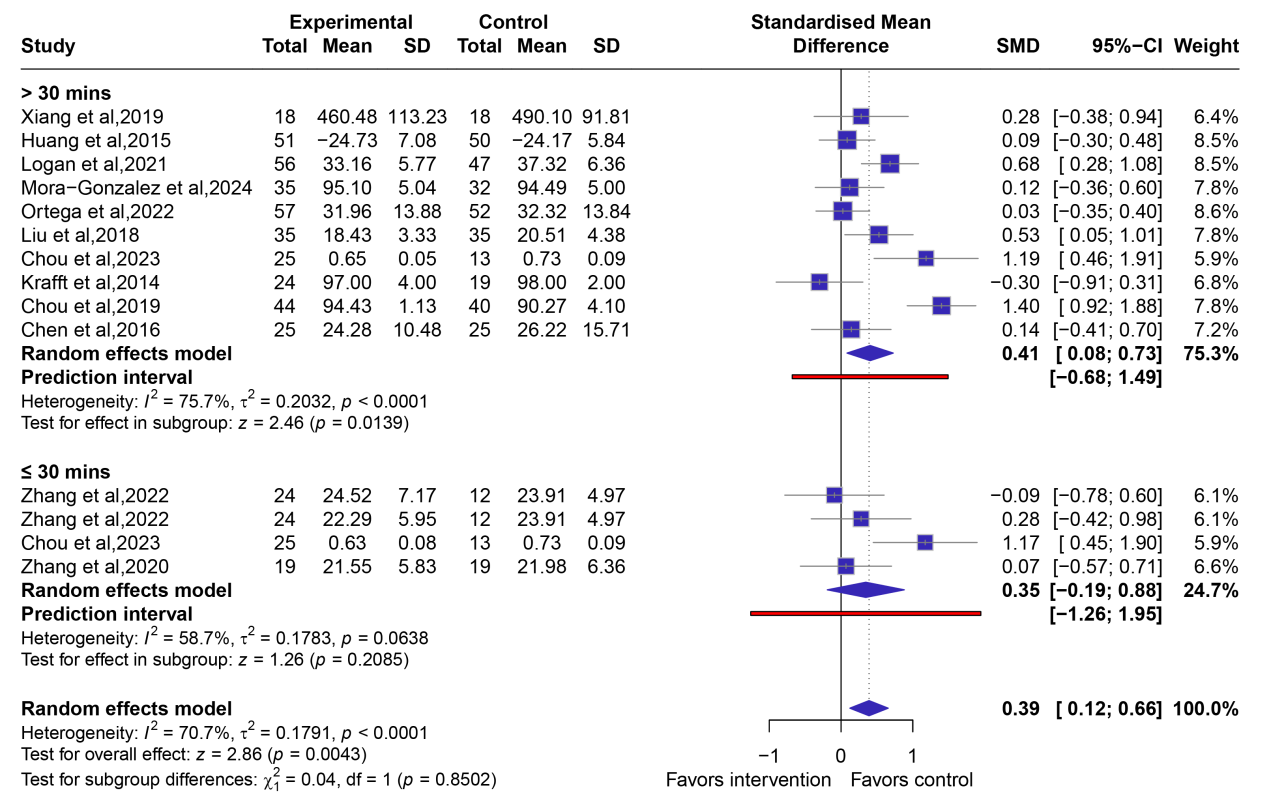


Figure S4. Subgroup analysis of session duration on **executive function**.

A positive effect value indicates a improvement in executive function. The effect size is Hedges’g.

Effe**cts of weekly exercise volume on executive function**

Subgroup analysis revealed a significant positive effect of weekly exercise volume > 120 mins on improving executive function (g: 0.28, 95%CI: 0.04, 0.53, P=0.0236), whereas weekly exercise volume ≤120 mins did not demonstrate a significant effect (g: 0.58, 95%CI: -0.02, 1.19, P=0.0595), as illustrated in Figure S5.


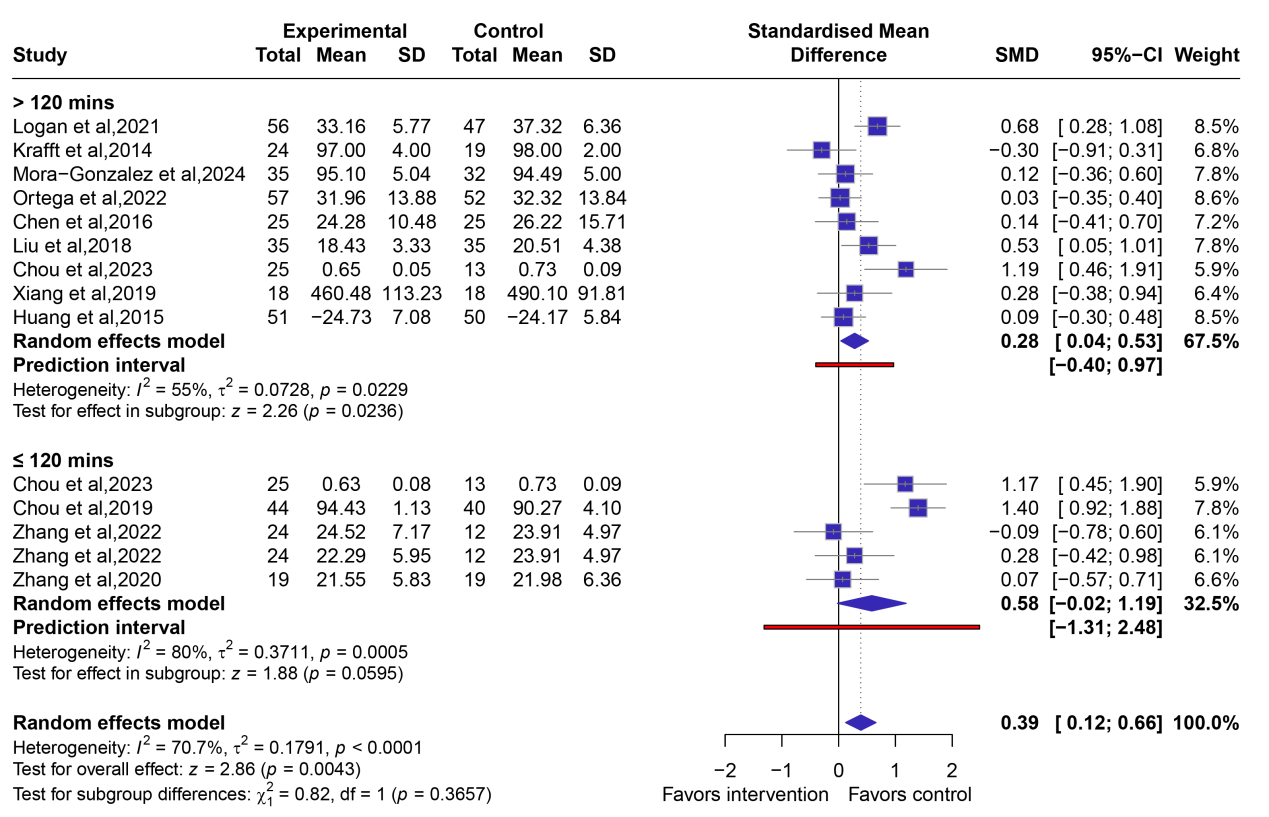


Figure S5. Subgroup analysis of weekly exercise volume on **executive function**.

A positive effect value indicates a improvement in executive function. The effect size is Hedges’g.

Effects of intervention period on **executive function**

Subgroup analysis revealed a significant positive effect of long-term (≥10 weeks) exercise on improving **executive function** (g: 0.41, 95%CI: 0.07, 0.75, P=0.0184), whereas short-term (<10 weeks) exercise did not demonstrate a significant effect (g: 0.36, 95%CI: -0.11, 0.82, P=0.1308), as illustrated in Figure S6.


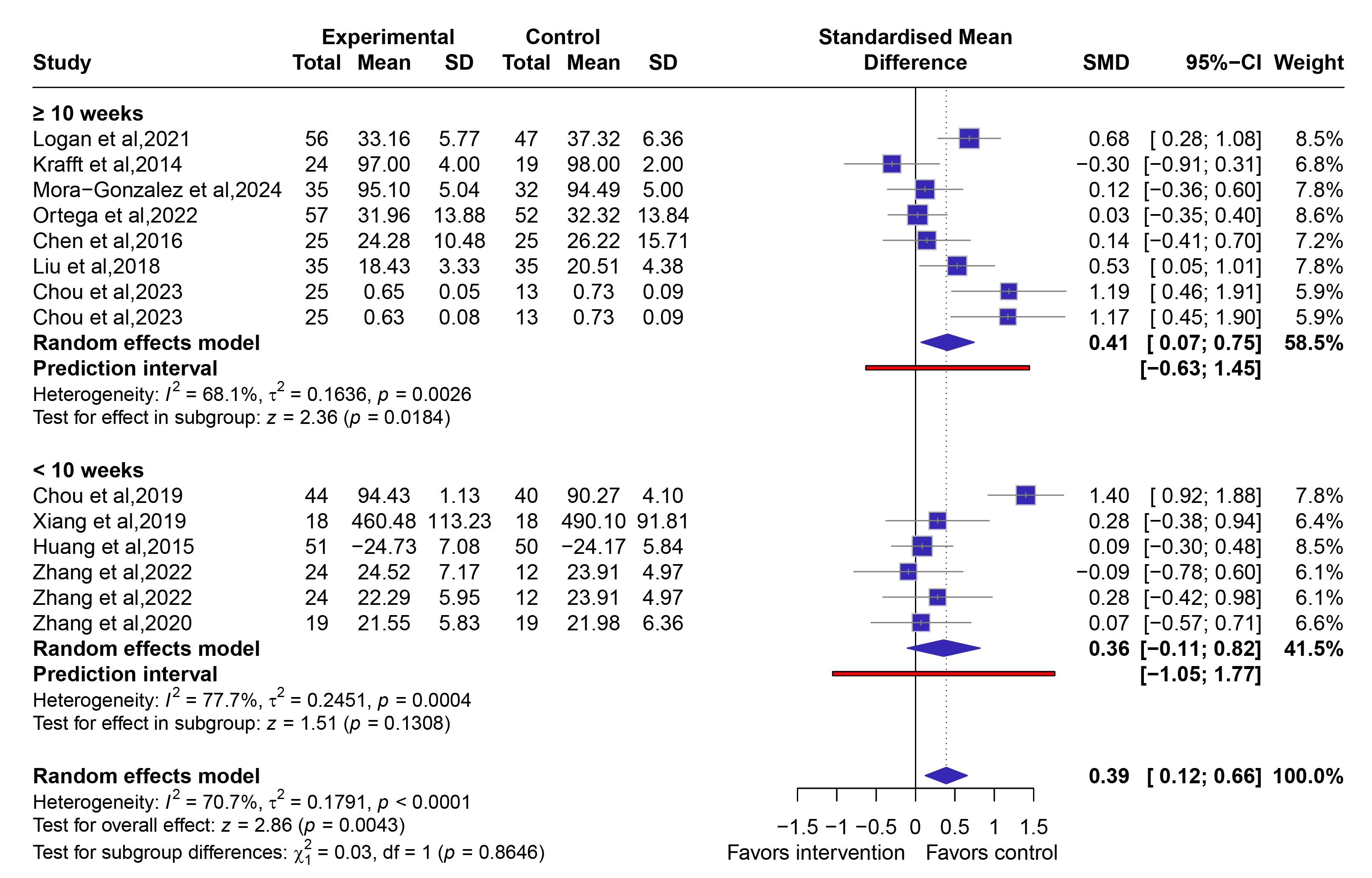


Figure S6. Subgroup analysis of intervention period on **executive function**.

A positive effect value indicates a improvement in **executive function**. The effect size is Hedges’g

Effects of age on **executive function**

**Subgroup analysis stratified by age revealed significant improvements in executive function in both children (g: 0.44, 95% CI: 0.07, 0.81, *p*=0.0186) and adolescents (g: 0.29, 95% CI: 0.01, 0.57, *p*=0.0448) , with a greater SMD observed in adolescents compared to children(Figure S7).**


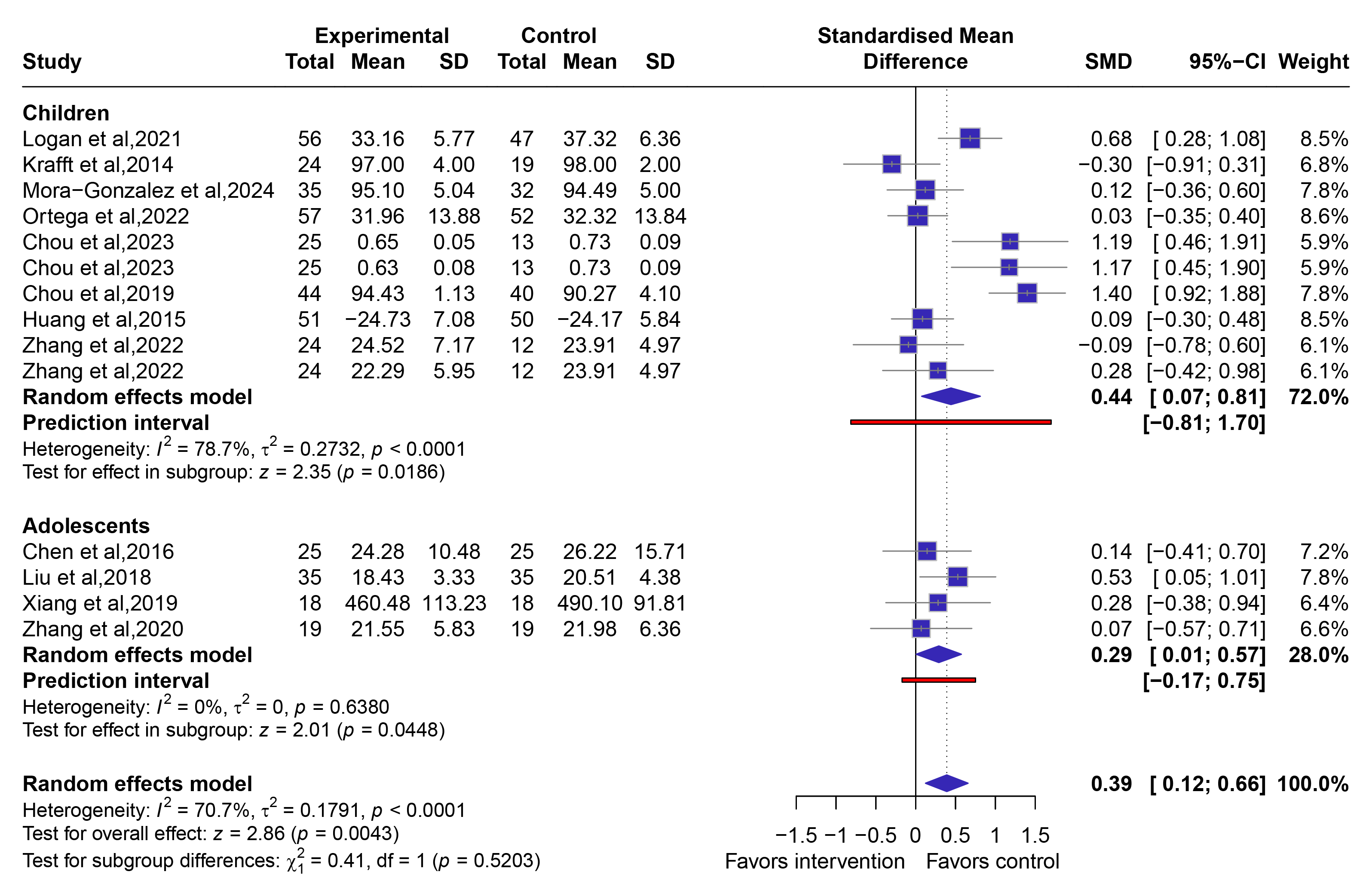


Figure S7. Subgroup analysis of age on **executive function**.

A positive effect value indicates a improvement in executive function. The effect size is Hedges’g.

Effects of BMI on **executive function**

**Subgroup analysis based on BMI demonstrated that overweight children and adolescents(g: 0.87, 95% CI: 0.37, 1.36, *p*=0.0006) exhibited significantly greater intervention-induced improvements in executive function compared to their obese counterparts (g: 0.13, 95% CI: -0.05, 0.31, *p*=0.1696) (Figure S8).**


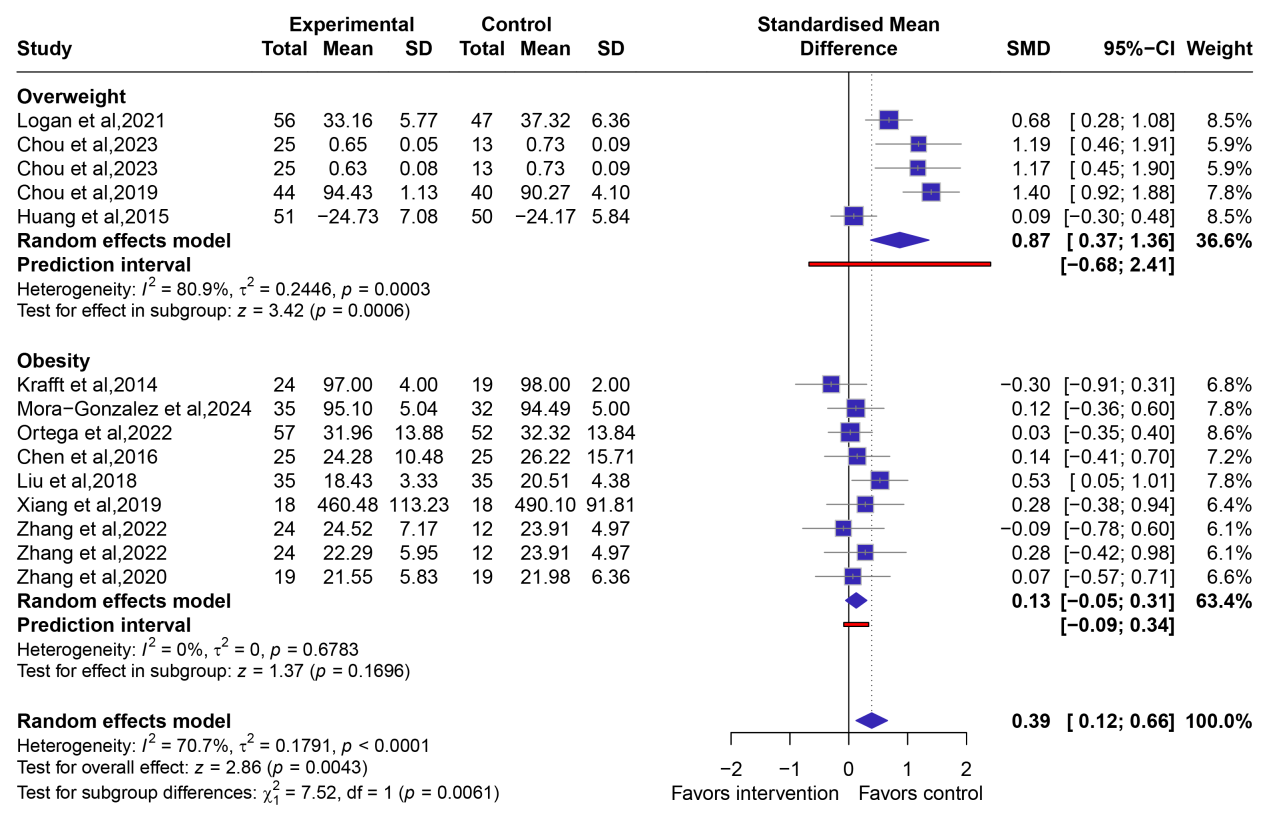


Figure S8. Subgroup analysis of BMI on **executive function**.

A positive effect value indicates a improvement in executive function. The effect size is Hedges’g.

Exercise Dose-Response Meta-Regression for Executive Function

Meta-regression exploring heterogeneity sources revealed no significant effects of exercise dose parameters (duration/volume/frequency/total time) on executive function(P > 0.05), with consistent but non-significant negative associations with SMD(Figure S9).


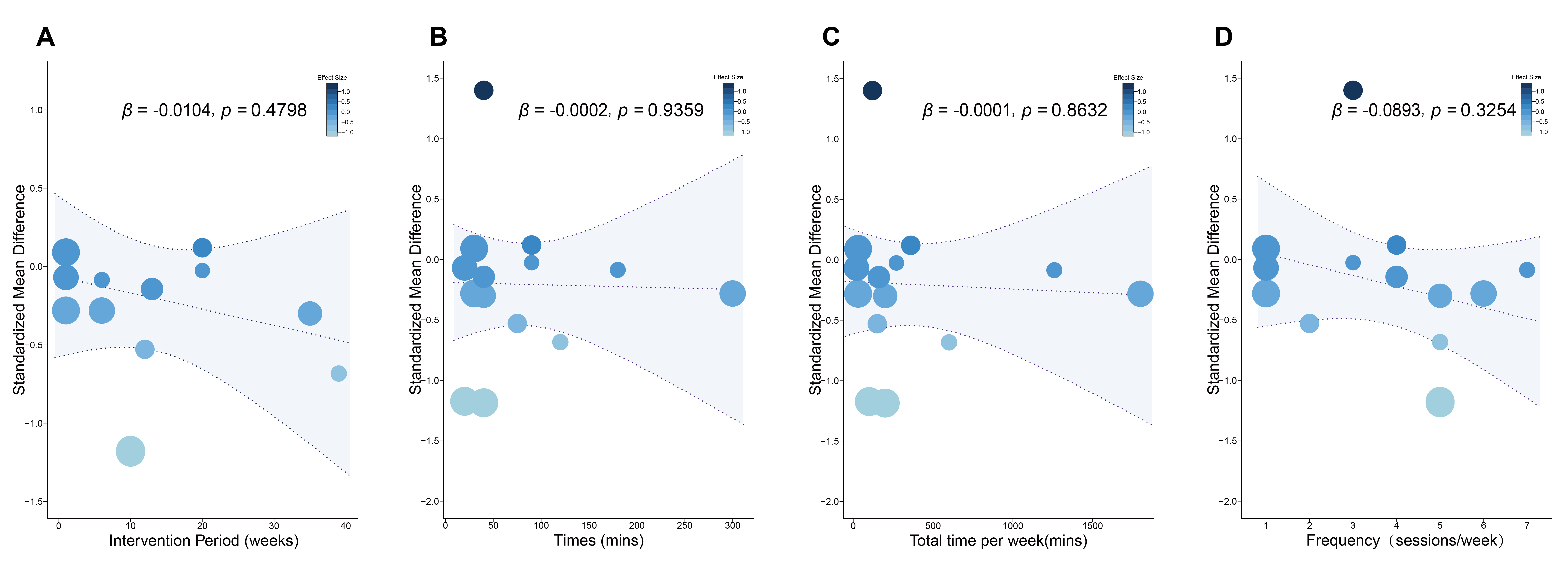


Figure S9. Regulatory effects of exercise on **executive function** and results of meta-regression analysis of training-related predictors


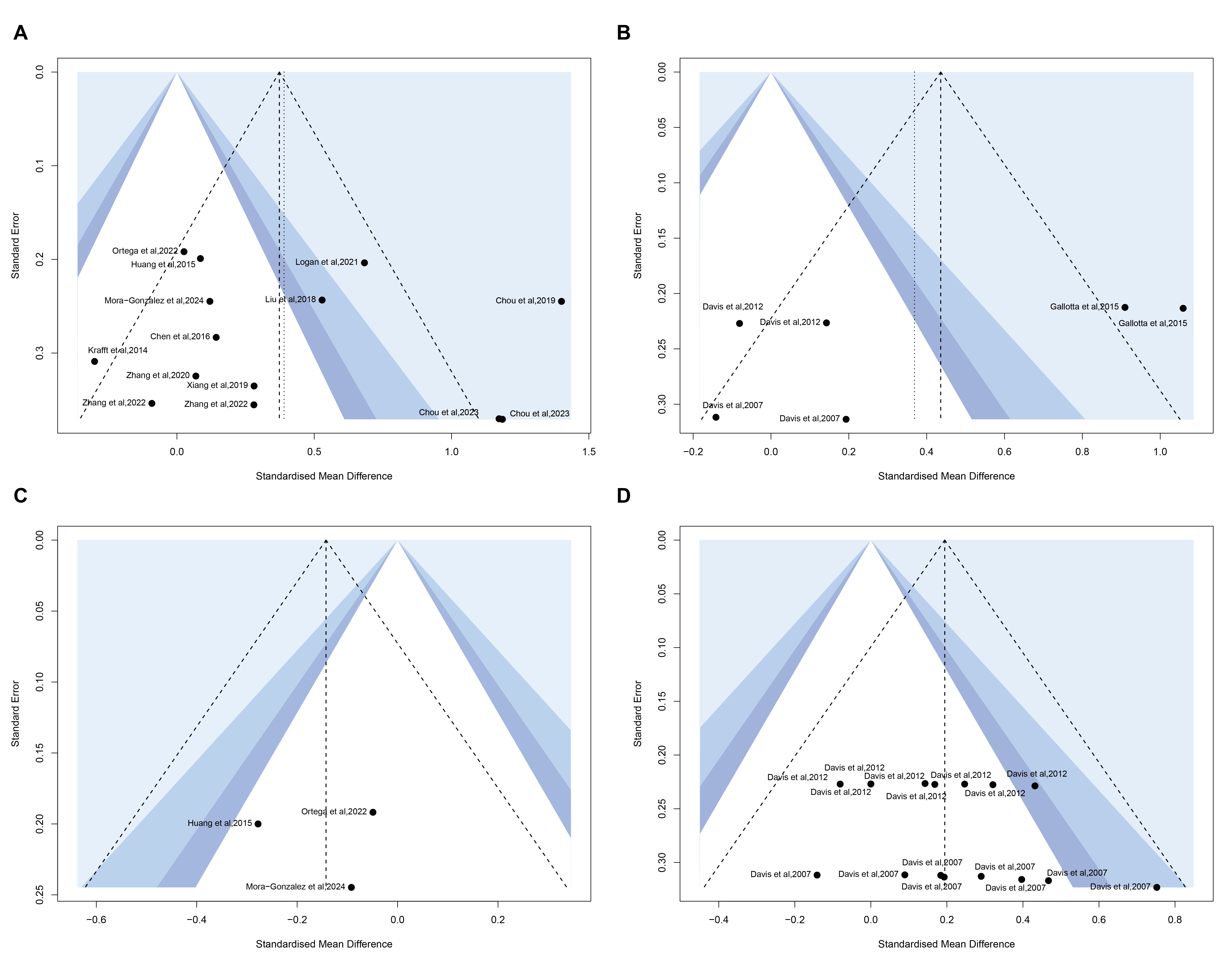


Figure. S10. Publishing bias funnel plots


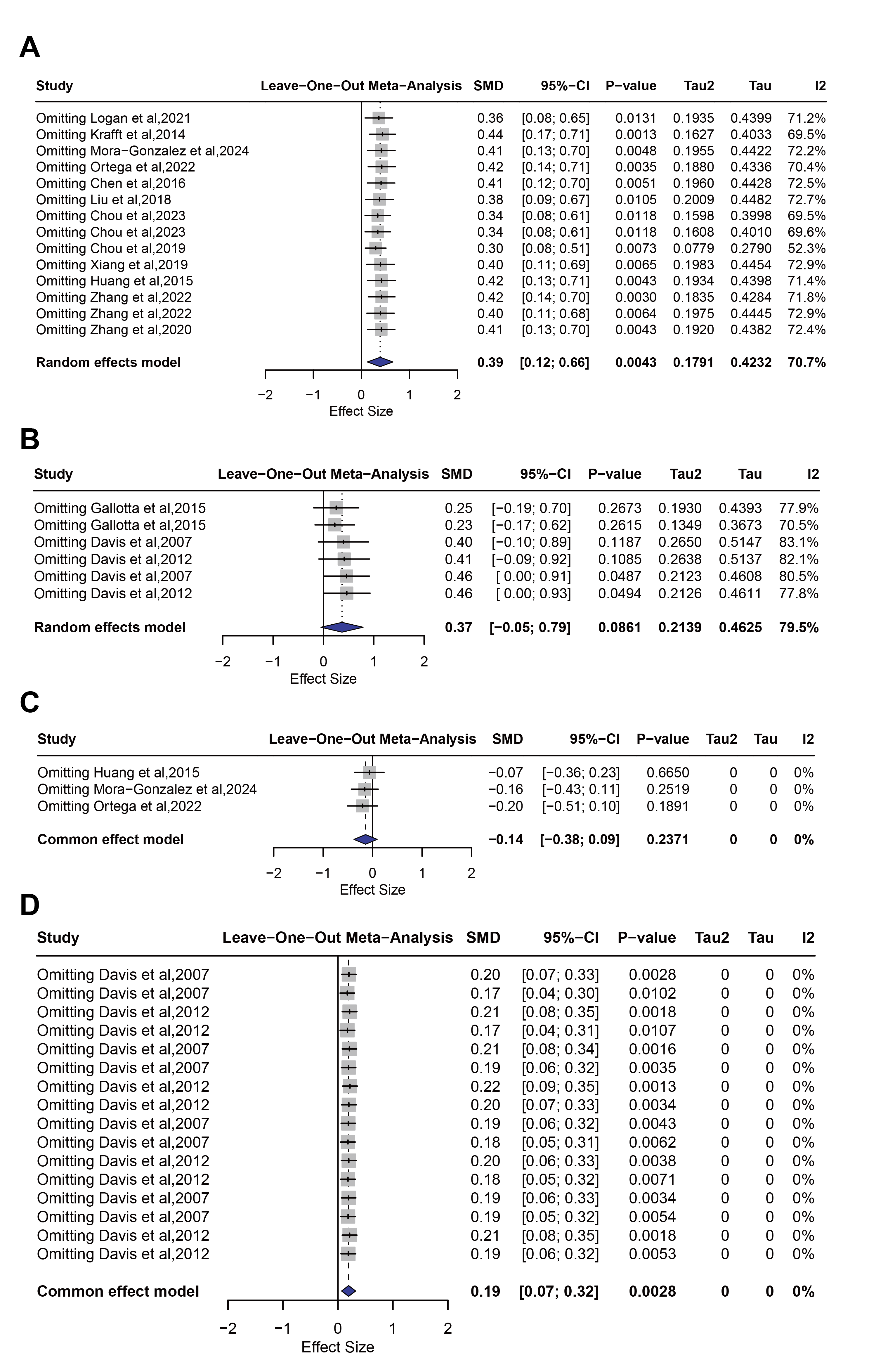


Figure S11. sensitivity analysis

**
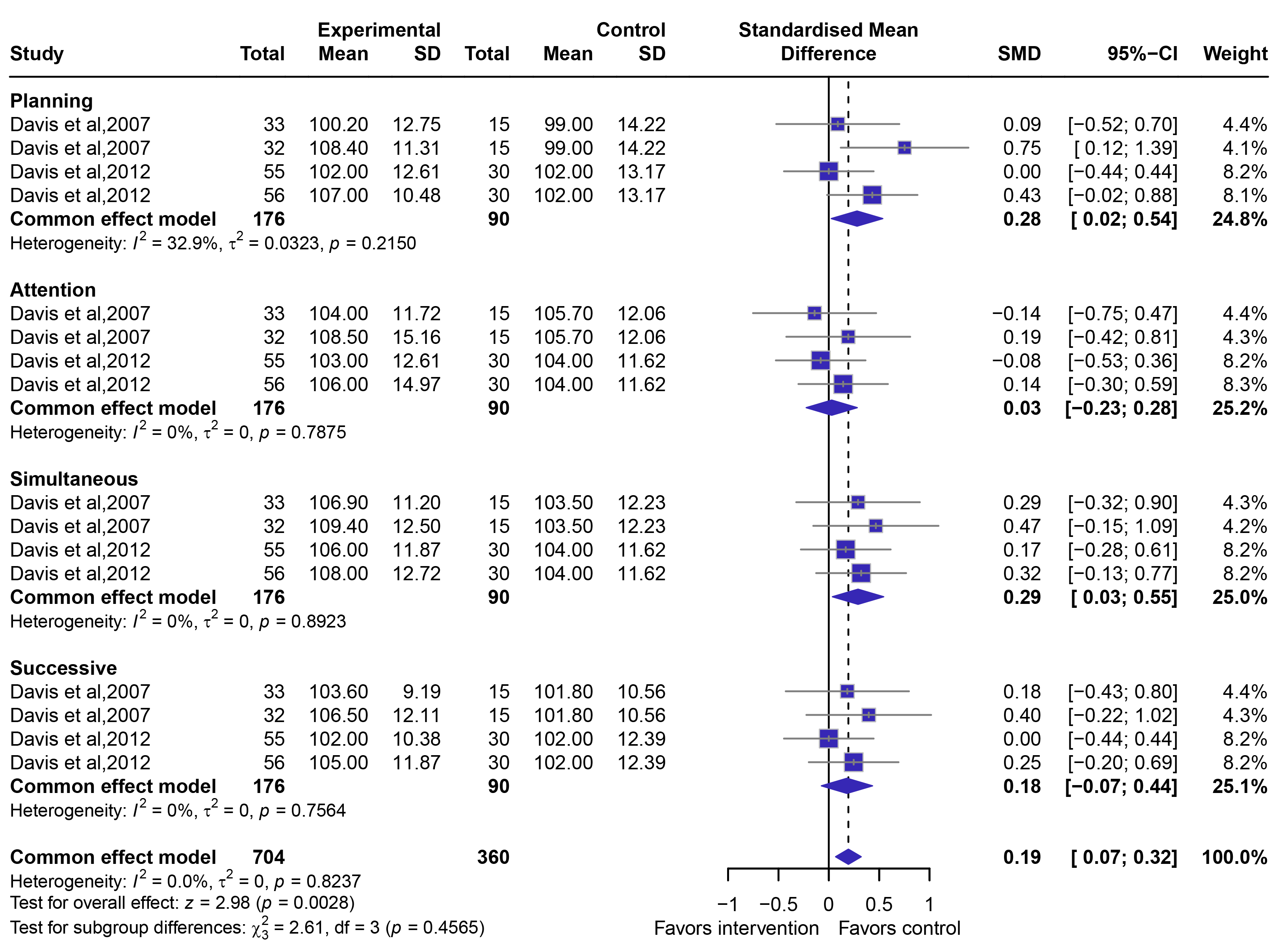
**

**Figure S12.** Forest plots of exercise intervention on **CAS-assessed executive function**

A positive effect value indicates a improvement in executive function. The effect size is Hedges’g.


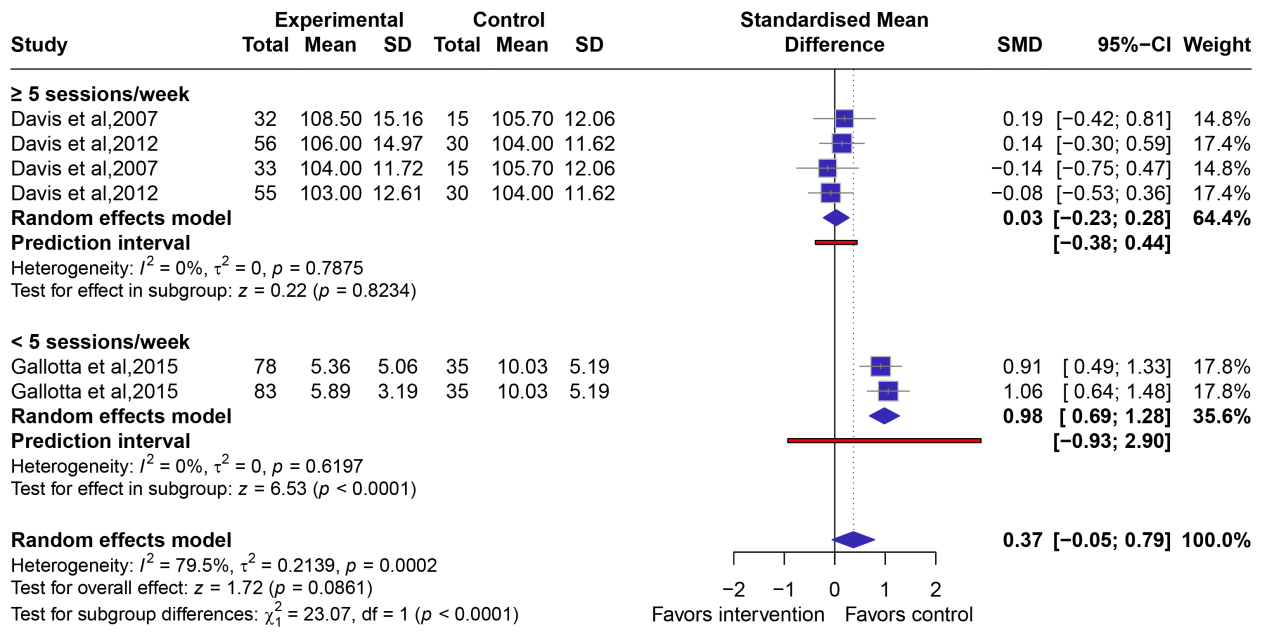


Figure S13. Subgroup analysis of exercise frequency on attention function

A positive effect value indicates a improvement in executive function. The effect size is Hedges’g.


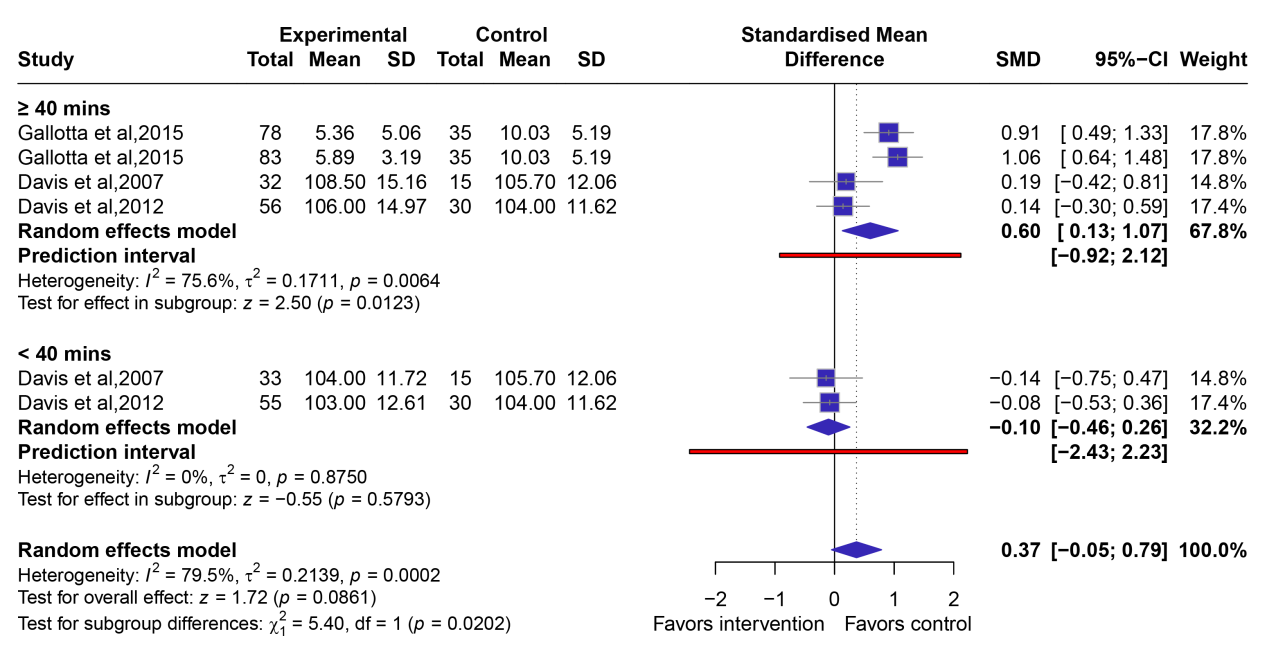


Figure S14. Subgroup analysis of session duration on attention function.

A positive effect value indicates a improvement in executive function. The effect size is Hedges’g.


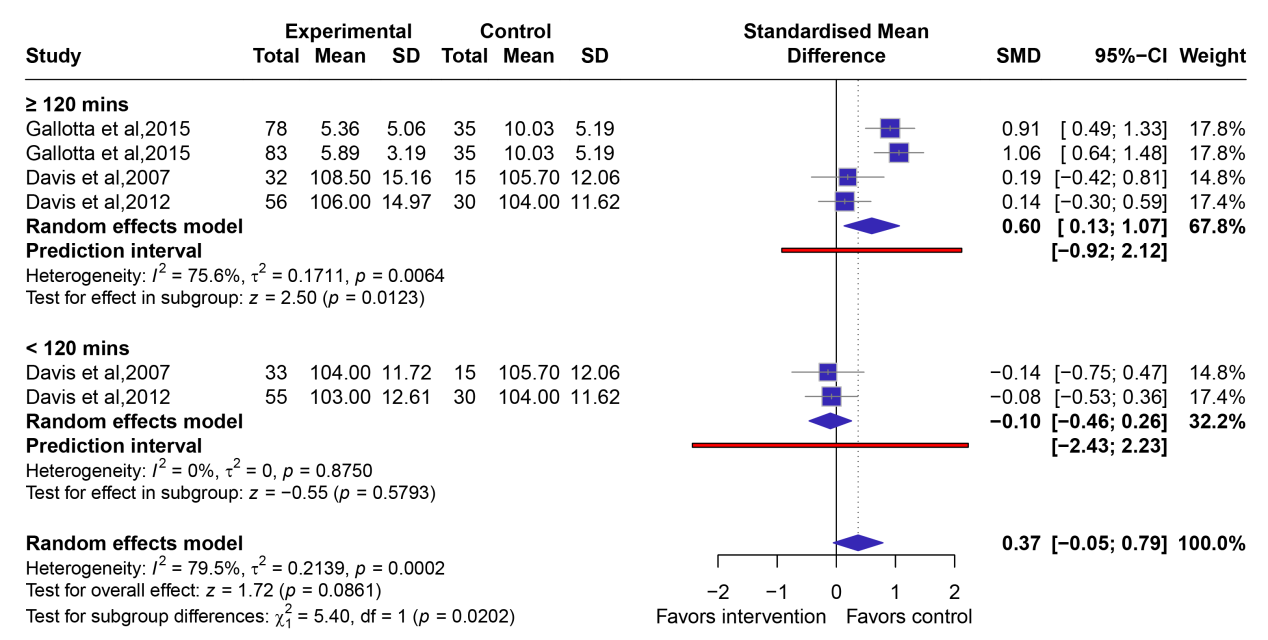


Figure S15. Subgroup analysis of weekly exercise volume on attention function.

A positive effect value indicates a improvement in executive function. The effect size is Hedges’g.


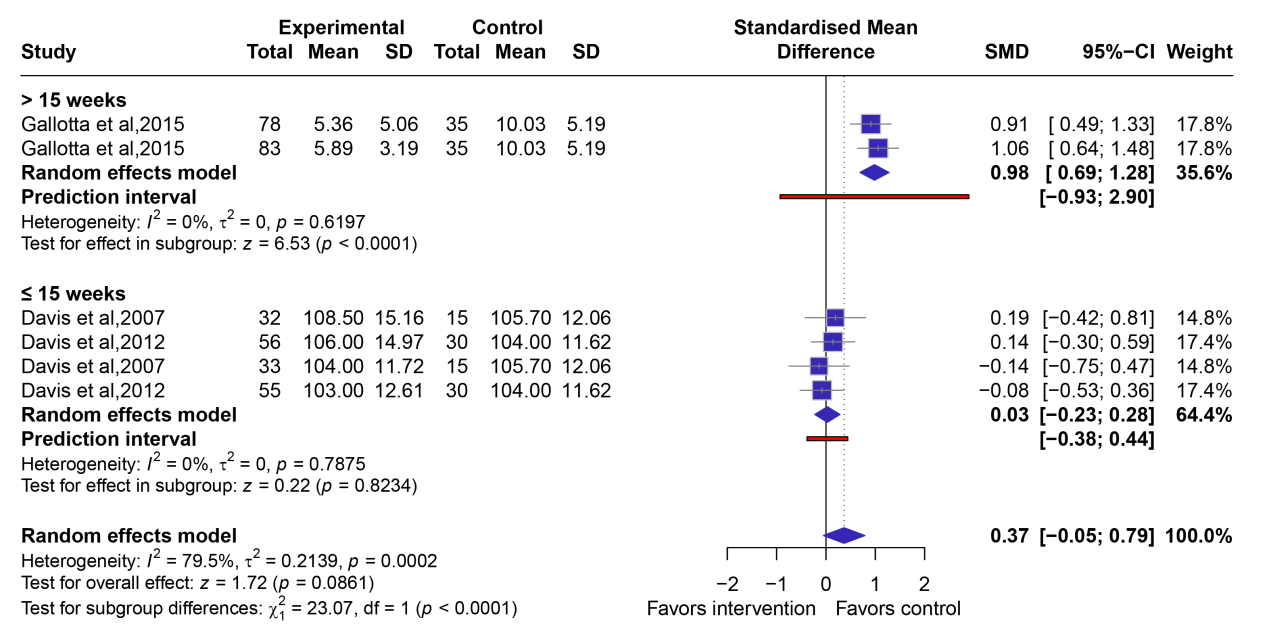


Figure S16. Subgroup analysis of intervention period on attention function..

A positive effect value indicates a improvement in **executive function**. The effect size is Hedges’g


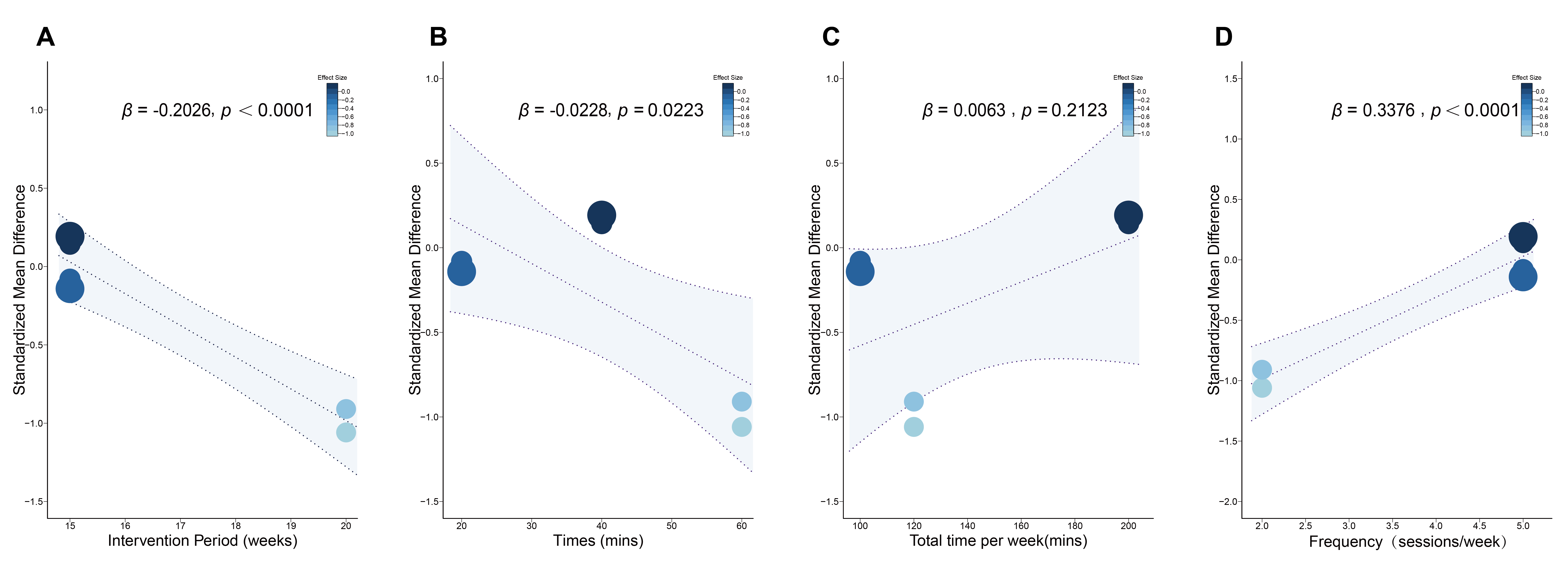


Figure S17. Regulatory effects of exercise on **attention function** and results of meta-regression analysis of training-related predictors


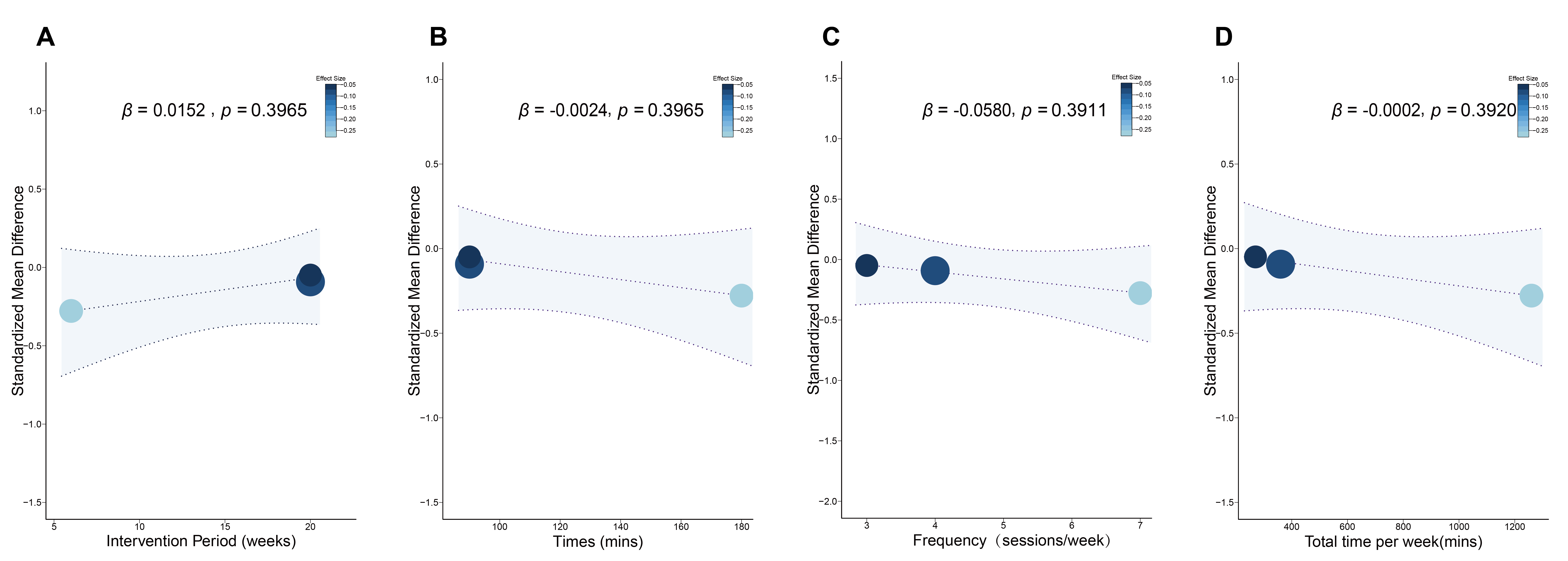


Figure S18. Regulatory effects of exercise on **memory function** and results of meta-regression analysis of training-related predictors
