# Supplementary material for: Effects of exercise on cognitive function in children and adolescents with overweight or obesity: a systematic review and meta-analysis of randomized controlled trials
Source: Front Public Health. 2025 Dec 12;13:1694170. doi: 10.3389/fpubh.2025.1694170 (PMC12741061; doi:10.3389/fpubh.2025.1694170)
Supplement: Supplementary file 2 [file Table_1.doc]

**Effects of exercise on cognitive function in overweight or obesity children and adolescents: A systematic review and meta-analysis of randomized controlled trials**

**Supplementary Material**

**1. Search Strategy**

****Pubmed****

((sport[Title/Abstract] OR physical activity[Title/Abstract] OR physical performance[Title/Abstract] OR physical fitness[Title/Abstract] OR physical exercise[Title/Abstract] OR physical exertion[Title/Abstract] OR physical intervention[Title/Abstract] OR physical training[Title/Abstract] OR aerobic exercise[Title/Abstract] OR nonaerobic exercise[Title/Abstract] OR exercise therapy[Title/Abstract] OR exercise intervention[Title/Abstract] OR exercise training[Title/Abstract] OR resistance training[Title/Abstract] OR endurance training[Title/Abstract] OR strength training[Title/Abstract] OR walk[Title/Abstract] OR running[Title/Abstract] OR jogging[Title/Abstract] OR hiking[Title/Abstract] OR cycling[Title/Abstract] OR bicycling[Title/Abstract] OR swim[Title/Abstract] OR aquatic[Title/Abstract] OR yoga[Title/Abstract] OR stretch[Title/Abstract] OR weight lift[Title/Abstract] OR exercise[Title/Abstract] OR training[Title/Abstract] OR mind-body exercise[Title/Abstract]**)) and ((obese[Title/Abstract]) OR (obesity[Title/Abstract]) (overweight[Title/Abstract])) and (cognition OR cognitive OR cognitive functioning OR neurocognitive OR cognitions OR information processing OR attention OR intelligence OR executive function OR learning OR processing speed OR working memory OR memory OR cognitive performance) AND (((((randomized controlled trial[Publication Type]) OR (controlled clinical trial[Publication Type])) OR (randomized[Title/Abstract])) OR (placebo[Title/Abstract])) OR (RCT[Title/Abstract]))**

****Web of science****

(sport OR physical activity OR physical performance OR physical fitness OR physical exercise OR physical exertion OR physical intervention OR physical training OR aerobic exercise OR exercise therapy OR exercise intervention OR exercise training OR resistance training OR endurance training OR strength training OR walk OR running OR jogging OR hiking OR cycling OR bicycling OR swim OR aquatic OR yoga OR exercise OR training OR mind-body exercise ) AND (**obese** OR **obesity** OR **overweight** ) AND **(cognition OR cognitive OR cognitive functioning OR neurocognitive OR cognitions OR information processing OR attention OR intelligence OR executive function OR learning OR processing speed OR working memory OR memory OR cognitive performance)** AND (((randomized controlled trial[Publication Type]) OR (controlled clinical trial[Publication Type] OR randomized[Title/Abstract])) OR (placebo[Title/Abstract])) OR (RCT[Title/Abstract])

**Embase**

(sport) OR (physical activity) OR( physical performance) OR (physical fitness) OR (physical exercise) OR (physical exertion) OR (physical intervention) OR( physical training) OR (aerobic exercise) OR (nonaerobic exercise) OR (exercise therapy) OR (exercise intervention) OR (exercise training) OR (resistance training) OR (endurance training) OR (strength training) OR (walk OR (running) OR( jogging) OR (hiking) OR (cycling) OR (bicycling) OR (swim) OR( aquatic) OR ( yoga) OR (exercise) OR (training) OR (mind-body exercise)

AND

(**obese**) OR (**obesity**) OR (**overweight**)

AND

(cognition) OR (cognitive) OR (cognitive functioning) OR (neurocognitive) OR cognitions) OR (information processing) OR (attention) OR (intelligence) OR (executive function) OR (learning OR (processing speed) OR (working memory) OR (memory) OR (cognitive performance)

1. Table S1 Methodological quality of the included studies

| Items | | | | | | | | | | | | | |
| --- | --- | --- | --- | --- | --- | --- | --- | --- | --- | --- | --- | --- | --- |
| Study | 1 | 2 | 3 | 4 | 5 | 6 | 7 | 8 | 9 | 10 | 11 | Score^a^ | Quality rating |
| Mora-Gonzalez J (2024) | 1 | 1 | 1 | 1 | 0 | 0 | 1 | 0 | 1 | 1 | 1 | 7 | High |
| Chou CC (2023) | 1 | 1 | 0 | 1 | 0 | 0 | 0 | 0 | 0 | 1 | 1 | 4 | Moderate |
| Ortega FB (2022) | 1 | 1 | 1 | 1 | 0 | 0 | 1 | 0 | 1 | 1 | 1 | 7 | High |
| Zhang L (2022) | 1 | 1 | 0 | 1 | 0 | 0 | 1 | 1 | 1 | 1 | 1 | 7 | High |
| Logan NE (2021) | 1 | 1 | 0 | 1 | 0 | 0 | 1 | 1 | 1 | 1 | 1 | 7 | High |
| Zhang L (2020) | 1 | 1 | 1 | 1 | 0 | 0 | 1 | 1 | 1 | 1 | 1 | 8 | High |
| Chou C (2019) | 1 | 1 | 0 | 1 | 0 | 0 | 1 | 1 | 0 | 1 | 1 | 6 | High |
| Xiang MQ (2019) | 1 | 1 | 0 | 1 | 0 | 0 | 0 | 0 | 0 | 1 | 1 | 4 | Moderate |
| Liu JH (2018) | 1 | 1 | 1 | 1 | 0 | 0 | 1 | 1 | 1 | 1 | 1 | 8 | High |
| Chen SR (2016) | 1 | 1 | 0 | 1 | 0 | 0 | 0 | 0 | 0 | 1 | 1 | 4 | Moderate |
| Huang T (2015) | 1 | 1 | 1 | 1 | 0 | 0 | 1 | 1 | 1 | 1 | 1 | 8 | High |
| Gallotta MC (2015) | 1 | 1 | 0 | 1 | 0 | 0 | 1 | 1 | 0 | 1 | 1 | 6 | High |
| Krafft CE (2014) | 1 | 1 | 1 | 1 | 0 | 0 | 1 | 0 | 1 | 1 | 1 | 7 | High |
| Davis CL (2012) | 1 | 1 | 1 | 1 | 0 | 0 | 1 | 1 | 1 | 1 | 1 | 8 | High |
| Davis CL (2007) | 1 | 1 | 0 | 1 | 0 | 0 | 1 | 1 | 1 | 1 | 1 | 7 | High |

Items: (1) eligibility criteria; (2) randomization; (3) concealed allocation; (4) similarity at baseline; (5) subjects blinding; (6) blinding therapists; (7) assessors blinding; (8) one key outcome measured in > 85% of subjects; (9) intention to treat; (10) between-group statistical results for one key outcome; (11) measures of variability and point measures for one key outcome. ^a^ The total score on a 10-point scale is calculated according to the number of criteria met, with the exception that scale item 1 is not included in the computation of the overall score.
